# Ablation of the presynaptic organizer Bassoon in excitatory neurons retards dentate gyrus maturation and enhances learning performance

**DOI:** 10.1007/s00429-018-1692-3

**Published:** 2018-06-18

**Authors:** Anil Annamneedi, Gürsel Caliskan, Sabrina Müller, Dirk Montag, Eike Budinger, Frank Angenstein, Anna Fejtova, Wolfgang Tischmeyer, Eckart D. Gundelfinger, Oliver Stork

**Affiliations:** 1 Department of Neurochemistry and Molecular Biology, Leibniz Institute for Neurobiology, Magdeburg, Germany; 2 Department of Genetics and Molecular Neurobiology, Institute of Biology, Otto-von-Guericke-University, Magdeburg, Germany; 3 Neurogenetics Laboratory, Leibniz Institute for Neurobiology, Magdeburg, Germany; 4 Department of Systems Physiology of Learning, Leibniz Institute for Neurobiology, Magdeburg, Germany; 5 Special Laboratory Noninvasive Brain Imaging, Leibniz Institute for Neurobiology, Magdeburg, Germany; 6 RG Presynaptic Plasticity, Leibniz Institute for Neurobiology, Magdeburg, Germany; 7 Center for Behavioral Brain Sciences (CBBS), Magdeburg, Germany; 8 Department of Psychiatry and Psychotherapy, University Hospital, Friedrich-Alexander-University Erlangen-Nuremberg, Erlangen, Germany; 9 Special Laboratory Molecular Biological Techniques, Leibniz Institute for Neurobiology, Magdeburg, Germany; 10 Molecular Neuroscience, Medical School, Otto von Guericke University, Magdeburg, Germany; 11 Functional Neuroimaging Group, German Center for Neurodegenerative Diseases, Magdeburg, Germany

**Keywords:** Bassoon, Contextual fear memory, Spatial memory, Immature DG, Neurogenesis, Knockout mice

## Abstract

Bassoon is a large scaffolding protein of the presynaptic active zone involved in the development of presynaptic terminals and in the regulation of neurotransmitter release at both excitatory and inhibitory brain synapses. Mice with constitutive ablation of the *Bassoon* (*Bsn*) gene display impaired presynaptic function, show sensory deficits and develop severe seizures. To specifically study the role of Bassoon at excitatory forebrain synapses and its relevance for control of behavior, we generated conditional knockout (*Bsn* cKO) mice by gene ablation through an *Emx1* promoter-driven Cre recombinase. In these animals, we confirm selective loss of Bassoon from glutamatergic neurons of the forebrain. Behavioral assessment revealed that, in comparison to wild-type littermates, *Bsn* cKO mice display selectively enhanced contextual fear memory and increased novelty preference in a spatial discrimination/pattern separation task. These changes are accompanied by an augmentation of baseline synaptic transmission at medial perforant path to dentate gyrus (DG) synapses, as indicated by increased ratios of field excitatory postsynaptic potential slope to fiber volley amplitude. At the structural level, an increased complexity of apical dendrites of DG granule cells can be detected in *Bsn* cKO mice. In addition, alterations in the expression of cellular maturation markers and a lack of age-dependent decrease in excitability between juvenile and adult *Bsn* cKO mice are observed. Our data suggest that expression of Bassoon in excitatory forebrain neurons is required for the normal maturation of the DG and important for spatial and contextual memory.

**Electronic supplementary material:**

The online version of this article (10.1007/s00429-018-1692-3) contains supplementary material, which is available to authorized users.

## Introduction

The formation and maturation of neuronal circuits in development and consequently processes of memory formation critically depend on the dynamic regulation of synaptic transmission and excitability. Multi-domain scaffolding proteins play essential roles in these processes by assembling and (re-)organizing proteinaceous cytomatrices on both sides of chemical synapses. In the presynaptic cytomatrix at the active zone (CAZ), which organizes the synaptic vesicle cycle underlying regulated neurotransmitter exocytosis, the two closely related proteins Bassoon and Piccolo/Aczonin fulfill such scaffolding functions together with other multi-domain proteins, such as the Rab3-interacting molecules (RIMs), RIM-binding proteins (RBPs), ELKS/CAST proteins, and Munc13s (Fejtova and Gundelfinger 2006; Schoch and Gundelfinger 2006; Südhof 2012; Gundelfinger and Fejtova 2012; Ackermann et al. 2015). Bassoon is a very large CAZ protein (420 kDa) involved in the developmental assembly of the active zones (Shapira et al. 2003; Ziv and Garner 2004; Maas et al. 2012) as well as in processes of presynaptic plasticity (for review see: Gundelfinger and Fejtova 2012; Ivanova et al. 2016). The *Bsn* gene has been well-conserved among mammals including humans (Winter et al. 1999). Bassoon expression is significantly up-regulated in the prefrontal cortex of schizophrenic patients (Martins-de-Souza et al. 2009) and the content of the protein in the synaptic proteome is rapidly down-regulated after frequency-modulated tone discrimination learning in mice (Kähne et al. 2012).

The analysis of constitutive *Bsn* mutant mice and of primary neuronal cultures derived from these mice has implied multiple critical synaptic functions for the protein. To date, two different *Bsn* mutants have been studied: *Bsn*^*ΔEx4*/*5*^, a hypomorphic mutant lacking exons 4 and 5 encoding a large central part of the Bassoon (Altrock et al. 2003), and *Bsn*^*gt*^, a gene trap mutant lacking Bassoon at most synapses in the brain (Hallermann et al. 2010; Frank et al. 2010). Both mutants display similar phenotypes with sensory disturbances and seizures. Bassoon is importantly involved in anchoring synaptic ribbons, i.e., highly specialized forms of the presynaptic cytomatrix, to the active zone. This has been observed at photoreceptor ribbon synapses (Dick et al. 2003; tom Dieck et al. 2005) as well as ribbon synapses of inner ear hair cells (Khimich et al. 2005; Frank et al. 2010) and likely accounts for deficits in visual and auditory sensory processing of Bassoon-mutant animals. Moreover, Bassoon is involved in the localization of presynaptic voltage-gated Ca^2+^ channels. This has been observed at inner ear hair cell synapses (Frank et al. 2010; Jing et al. 2013) as well as at excitatory hippocampal synapses, where specifically P/Q-type Ca^2+^ channels are mislocalized (Davydova et al. 2014). Bassoon-deficient synapses show a deficit in the replenishment of synaptic vesicles, most obviously at synapses with particularly high firing rates such as the cerebellar mossy fiber synapse or the endbulb of Held in the auditory system (Hallermann et al. 2010; Mendoza Schulz et al. 2014). Bassoon and its paralog Piccolo are involved in the maintenance of synaptic integrity by regulating presynaptic ubiquitination and proteostasis (Waites et al. 2013). The loss of these two proteins triggers degradation of synaptic vesicles and enhances autophagocytic processes in presynaptic terminals. Essentially, the interplay of Bassoon with Atg5, an E3-like ubiquitin ligase crucial for autophagy, is critical for the control of the formation of autophagic structures in presynaptic boutons (Okerlund et al. 2017). Another important function that is shared by Bassoon and Piccolo is the activity-dependent recruitment of the transcriptional suppressor protein CtBP1 to presynapses (Ivanova et al. 2015). In this way they control the distribution of CtBP1 between synapses and nuclei within neurons and in turn the expression of CtBP1-dependent genes (Ivanova et al. 2015, 2016; Gundelfinger et al. 2016). For example, Bassoon-deficient synapses recruit 30–40% less CtBP1 and display an altered synapto-nuclear distribution of the transcriptional repressor (Ivanova et al. 2015).

Another peculiar phenotype of *Bsn*^*ΔEx4*/*5*^ mutant mice is their increase in forebrain volume (Angenstein et al. 2007) that starts to become significant one month after birth and is accompanied by increased levels of brain-derived growth factor (BDNF; Heyden et al. 2011). *Bsn*^*ΔEx4*/*5*^ mice moreover display reduced synaptic fatigue during induction of long-term depression (LTD), spontaneous epileptic seizures (Altrock et al. 2003), impaired long-term potentiation (LTP) at CA1 synapses (Sgobio et al. 2010) and an abnormal synaptic plasticity at various striatal synapses (Ghiglieri et al. 2009). In the hippocampus, these animals further display an increased neurogenesis, reduced apoptosis (Heyden et al. 2011) and disturbance in the development of mossy fiber synapses (Lanore et al. 2010).

These data demonstrate that Bassoon is of critical importance for a variety of synaptic and network functions throughout the central nervous system. However, an in-depth behavioral assessment of the consequences of their disturbance in *Bsn*^*ΔEx4*/*5*^ mice is hampered by their visual and auditory impairment (Dick et al. 2003; Khimich et al. 2005) and the development of epilepsy (Altrock et al. 2003). In initial experiments, an altered performance in a socially transmitted food preference task (Sgobio et al. 2010) and an improved performance in a two-way active avoidance task that could be normalized by a TrkB antagonist (Ghiglieri et al. 2010) were observed. However, the underlying cellular processes are difficult to address due to above-mentioned sensory impairments and potential gain of function effects by the residual Bassoon fragment lacking its central part, i.e., about two-thirds of the entire protein (Altrock et al. 2003). Furthermore, it has to be considered that Bassoon is expressed at both excitatory and inhibitory neurons (Richter et al. 1999).

To address Bassoon functions in different types of neurons and their role for behavioral control, we have begun to dissect its functions genetically. Here, we report the generation and characterization of mice conditionally lacking Bassoon at glutamatergic synapses of the forebrain (hippocampus and neocortex). We characterized these animals behaviorally and addressed the putative physiological and morphological correlates of the observed disturbances. Based on the above-mentioned disturbances of hippocampal functions in constitutive *Bsn* mutants, we focused our analysis mainly on hippocampus-dependent behavior and memory formation. In fact, our data thus reveal an altered performance of *Bsn* cKO mice in contextual and spatial discrimination/pattern separation task, associated with increased excitability at the medial perforant path (MPP) to dentate gyrus (DG) synapse and morphological and physiological changes in DG granule cells that are indicative of a reduced maturation of the DG and increased adult neurogenesis in these animals.

## Materials and methods

### Antibodies

Primary antibodies raised in mouse include anti-Bassoon (mab7f catalog #ADI-VAM-PS003-F, Enzo Life Sciences Inc, New York, USA, RRID:AB_10618753) (1:1000, Immunohistochemistry (IHC) and Western blotting (WB)) and anti-Tubulin-β (#T8660, Sigma-Aldrich, Missouri, USA, RRID:AB_477590) (1:1000, WB). Antibodies raised in rabbit include anti-Bassoon (tom Dieck et al. 1998; homemade, RRID:AB_2313989) (1:1000, WB), anti-Calbindin (#CB 38a, Swant, Marly1, Switzerland, RRID:AB_10000340) (1:1500, IHC), anti-Calretinin (#CR 7697, Swant, RRID:AB_2619710) (1:1250, IHC), anti-Ki67 (#ab15580, Abcam, Cambridge, UK, RRID:AB_443209) (1:500, IHC), anti-Vesicular GABA transporter, VGAT (#131 002, Synaptic systems GmbH, Göttingen, Germany, RRID:AB_887871) (1:500, IHC) and anti-Vesicular glutamate transporter1, VGLUT1 (#135 302, Synaptic systems, RRID:AB_887877) (1:500, IHC). Other antibodies include goat anti-Doublecortin (C-18) (#sc-8066, Santa Cruz Biotechnology Inc, Dallas, USA, RRID:AB_2088494) (1:100, IHC). Fluorescent secondary antibodies raised in donkey include, anti-mouse Alexa 488 (#A21202, Invitrogen, California, USA, RRID:AB_141607) (1:500, IHC), anti-mouse Cy3 (#715-165-151, Jackson Immuno Research Labs, Pennsylvania, USA, RRID:AB_2315777) (1:500, IHC), anti-rabbit Alexa 488 (#A21206, Invitrogen, RRID:AB_141708) (1:500, IHC), anti-rabbit Cy3 (#711-165-152, Jackson Immuno Research, RRID:AB_2307443) (1:500, IHC), and anti-goat Cy3 (#705-165-147, Jackson Immuno Research, RRID:AB_2307351) (1:250, IHC). Antibodies raised in goat include anti-rabbit Alexa 680 (#A21109, Invitrogen, RRID:AB_2535758) and anti-mouse CF770 (#20077, Biotium, Inc. California, USA, RRID:AB_ 10559194) (1:20000, WB).

### Animals

All experiments were conducted in accordance with the European and German regulations for animal experiments and were approved by Landesverwaltungsamt Sachsen-Anhalt (Number: 42502-2-988 LIN and 42502-2-1303 LIN). Male *Bsn* cKO mice (see below) and wild-type (WT) littermates aged between 8 and 13 weeks were used for all experiments except for electrophysiology where young (27–33 days old) mice were also used.

### Generation of conditional knockout of Bassoon mice and genotyping details

To generate *Bsn* cKO mice lacking Bassoon at excitatory forebrain synapses, *Bsn2*^*lx*/*lx*^ mice were crossed with mice expressing Cre recombinase under the control of *empty spiracle homeobox-1* (*Emx1*) promoter (B6.129S2-Emx1^tm1(cre)Krj^, The Jackson Laboratory, Gorski et al. 2002). In *Bsn2*^*lx*/*lx*^ mice, loxP sites were inserted flanking exon 2 of the *Bsn* gene (Taconic Artemis GmbH, Germany; see Online Resource 1a, b). To this end, a targeting vector containing loxP sites on both sides of exon 2 along with a flippase recognition target (FRT)-flanked neomycin resistance gene (NeoR) in intron 1 and a F3-flanked puromycin resistance gene (PuroR) in intron 2 was constructed and used for homologous recombination and to select positive clones. The targeting vector was generated using clones from the C57BL/6J RPCIB-731 BAC library and transfected into Taconic Artemis C57BL/6 Tac embryonic stem cell line. The *Bsn2*^*lx*/*lx*^ cKO allele was obtained after Flp recombinase-mediated removal of Neo and Puro resistance genes. It can act as a substrate for Cre-mediated recombination. To generate *Bsn* cKO mice (*Bsn2*^*lx*/*lx*^/*Emx1*^*Cre*/+^), homozygous *Bsn2*^*lx*/*lx*^ mice were bred with *Emx1*-Cre driver mice (*B6.129S2-Emx1*^*tm1*(*cre*)*Krj*^) resulting in removal of exon 2 in principal glutamatergic forebrain neurons. The recombination leads to deletion of the N-terminal part of Bassoon’s 1st Zinc finger domain and causes a frameshift and the generation of a premature stop codon when spliced to any of following exons (tom Dieck et al. 1998; Winter et al. 1999; Online Resource 1c). Both *Bsn2*^*lx*/*lx*^ and *Emx1*-Cre driver lines were backcrossed to C57BL/6NCrl for at least 10 generations. Breeding was done at Leibniz Institute for Neurobiology, Magdeburg, and mice were maintained at 22 ± 2 °C, 12 h light–dark cycle, with lights on at 06:00 a.m., and with food and water ad libitum. Littermate experimental animals were obtained from *Bsn2*^*lx*/*lx*^*Emx1*^*Cre*/+^ × *Bsn2*^*lx*/*lx*^*Emx1*^+/+^ breedings. Genotyping for the *Bsn2*^*lx*/*lx*^ mice was done with polymerase chain reaction (PCR) using the forward primer (GCAGATTCTAGTCGGTGATCTAGC), reverse primer (GTTGCCTAATGTATGCAGAGTCC) and One Taq polymerase (New England BioLabs Inc, catalog#M0480X). The PCR protocol included an initial denaturation for 3 min at 95 °C followed by 35 cycles of 30 s denaturation at 95 °C, 30 s annealing at 60 °C, and 30 s synthesis at 68 °C, with final synthesis phase of 5 min at 68 °C. Thereby, a 220 bp wild-type (WT) allele could be discriminated from a 337 bp allele carrying the loxP sites (lox). Genotyping for Emx1-Cre was done as described by the supplier using One Taq polymerase and a PCR program 3 min at 94 °C, 35 × (30 s at 94 °C, 45 s at 62.3 °C, 45 s at 68 °C) and final 5 min at 68 °C.

### Immunohistochemistry

Mice were anesthetized with isoflurane and perfused transcardially with phosphate-buffered saline (PBS) followed by 4% paraformaldehyde (PFA). Brains were post-fixed overnight in the same fixative at 4 °C and cryoprotected by incubating them in 0.5 M sucrose in PBS and then in 1 M sucrose in PBS. Brains were then frozen using isopentane cooled by liquid nitrogen and stored at − 80 °C. Brains were transferred to − 20 °C the day before sectioning and 30–40 µm thick sagittal or coronal sections were cut on a cryostat, collected free floating, and used for immunological stainings or stored in a cryoprotection solution at − 20 °C until utilized. Immunohistochemical staining was done as described previously (Hubler et al. 2012) using antibodies listed above. For doublecortin (DCX) and calretinin immunohistochemistry, free-floating sections were first washed with PBS (10–15 min) and then incubated with blocking solution (5% bovine serum albumin (BSA) and 0.3% Triton X-100 in PBS) for 1 h at room temperature. Sections were incubated in primary antibody solution (2% BSA, 0.1% Triton X-100 in PBS), overnight at 4 °C. Later, the sections were washed in PBS (three times, 10 min each) followed by overnight incubation at 4 °C with appropriate secondary antibodies diluted in the same incubating solution as the respective primary antibody. Thereafter, the sections were washed with PBS (three times, 10 min each), mounted on glass slides and covered with coverslips using fluoromount g or fluoromount g DAPI (Southern biotech, USA) to protect them from bleaching and for nuclear counterstaining, respectively.

Overview micrographs of single sagittal brain sections were obtained using a Zeiss Axio Imager light microscope. Images were acquired in blocks (covering the entire section), using a 2.5× objective and arranged using Adobe InDesign CS6. For quantification of dentate gyrus (DG) maturation markers, every 5th section (i.e., each 150 µm apart) from dorsal DG (− 1.34 to − 2.46 mm from Bregma) was stained as described above. A total of 4–6 coronal sections covering the dorsal DG were analyzed per mouse (*N* = 5–6 mice per genotype). Confocal stacks of 0.65 µm Z-step size (~ 12 µm Z-stack volume) were taken with a Leica SP5 confocal microscope using 40× oil immersion objective (1.25–0.75 NA) and LCS software (Leica, Wetzlar, Germany) in the supragranular layer of DG. Maximal projection images from each stack were obtained using the Z-project function in Image-J software (version 1.50i, National Institutes of Health, http://rsb.info.nih.gov/ij/). The granule cell layer was marked (region of interest—ROI) based on DAPI staining using the free hand tool in Image-J. The same ROI was transferred to other channels for the marker analysis. Cell numbers were counted manually using the Cell Counter plugin and integrated density values were measured in the respective channel using Image-J. Overlapping cells were always rechecked using DAPI counter staining, to avoid chances of miscounting. Cell numbers were expressed as cells per 100 µm^2^ and integrated density (ID) values were normalized to mean of WT values. To assess adult neurogenesis, Ki67 expression was investigated in the DG as described in Clelland et al. 2009, with slight modifications. A Leica microscope (40×/0.75 NA objective) with motorized stage was used to track the granule cell layer using DAPI labeling and Ki67 positive cells were traced and marked along the rostro-caudal axis throughout the sections spanning dorsal DG. A total of 5 coronal sections from the dorsal DG were analyzed per mouse (*N* = 5 mice per genotype). Neurolucida software (MBF Bioscience) was used to reconstruct the images and Ki67 positive cell numbers were analyzed using the marker analysis tool in Neuroexplorer software (MBF Bioscience).

### Quantitative immunoblot analysis

Quantitative Western blotting was performed as described previously (Altrock et al. 2003; Lazarevic et al. 2011). Briefly, mice were killed by cervical dislocation and forebrain (containing cerebral cortex and hippocampus) and cerebellum were dissected from the brain. The tissue samples were homogenized in a buffer containing 0.32 M sucrose and 2.5 mM Tris–HCl (pH 7.4) supplemented with complete Protease Inhibitor (Roche) and PhosSTOP Phosphatase Inhibitor Cocktail (Roche) at 4 °C, resulting in homogenate fraction. The concentration of proteins was determined using a colorimetric Amido black (Serva Feinbiochemica GmbH, Heidelberg, Germany) assay and 10 µg protein per lane were loaded onto Tris–Acetate polyacrylamide gradient gels (8–4%) and ran at 10 mA per gel before being transferred onto Immobilon-FL PVDF membranes (Millipore). Blots were then incubated with primary antibodies (in PBS containing 5% BSA, 0.1% Tween and 0.025% sodium azide) at 4 °C overnight and with secondary antibodies (in PBS containing 1% BSA, 0.1% Tween) either at 4 °C overnight or at 1.5–2 h at room temperature. For quantification of signals, a measurement of integrated fluorescence densities (ID) was performed using an Odyssey Infrared Scanner (LI-COR). Identical rectangular ROIs were set around the bands to measure the ID values and values were normalized to loading controls and to the mean value of the WT group for each individual membrane.

### Morphological analysis

Morphological characteristics of different hippocampal neurons were analyzed using the Golgi impregnation method as described previously (Mylius et al. 2013; Rehberg et al. 2014). Granule cells of the dorsal DG and pyramidal neurons at the dorsal CA1 region of the hippocampus were analyzed using a light microscope (Leica, 100× objective) with motorized stage. Neuronal tracking and reconstruction were done using Neurolucida software (MBF Bioscience). Quantitative measurements of dendrite length and complexity were done using the Sholl analysis method (with 10 µm increasing radius from the center of the soma) tool in Neuroexplorer software (MBF Bioscience). The analysis was limited to a proximal proportion of the dendritic tree 0–120 µm from soma, to ensure a consistent reconstruction of Golgi impregnated structures in our preparations and to cover the inner and medial molecular layer of the DG with the relevant terminals of the MPP studied in our electrophysiological experiments (Amaral et al. 2007; Forster et al. 2006).

Analysis of total brain volume and regions like hippocampus, cortex and cerebellum volume analysis from WT and *Bsn* cKO mice was performed using Manganese-enhanced magnetic resonance imaging (ME-MRI) method as described previously (Angenstein et al. 2007; Heyden et al. 2011). Briefly, volumes were measured using the public domain Java-based image processing and analysis program Image-J and individual structures were manually segmented in each section. Because MRI measurements were performed on anesthetized living mice, the depicted brain anatomy represents the in vivo condition, means no shrinkage or any other deformation of the brain.

### Behavioral experiments

Male *Bsn* cKO mice and WT littermates were obtained at an age between 5 and 7 weeks (animal facility, Leibniz Institute for Neurobiology, Magdeburg) and transferred to Institute of Biology, Otto von Guericke University, Magdeburg, where most of the behavioral experiments were performed (except for Morris water maze, which was done in Leibniz Institute for Neurobiology, with a separate batch of mice). After transfer, mice were habituated for at least one week in individual cages under a reverse 12 h light/12 h dark cycle, lights on at 7 p.m., room temperature (22 ± 2 °C). All the experiments were performed between 9:00 a.m and 5:00 p.m and in different test batteries. Test battery one included home cage activity monitoring, light–dark test, open field, novel object location and fear conditioning. Test battery two included open field, novel object location and active avoidance. Test battery three included home cage activity monitoring and spatial discrimination/pattern separation. Care was taken to arrange tests such that interferences were avoided and sufficient recovery time was allowed. General and neurological assessment of the WT and cKO mice were done as described previously (Whishaw et al. 1999) and did not reveal any differences between the genotypes.

#### Home cage activity monitoring

As previously described (Bergado-Acosta et al. 2014), mice were monitored for four consecutive days in their home cages. Activity was measured using infrared-thermo sensors (Home Cage Activity System, Coulbourn Instruments, Allentown PA), mounted on the top of each cage and interfaced with a computer. Activity was determined from raw values (measured as movement between lower limit of 100 ms and upper limit of 500 ms) of 15 s, which were used to calculate activity periods of 5 min bins. Activity periods per hour were calculated from average values of 4 days.

#### Light–dark test

After 4 days observation of the home cage activity, anxiety-like behavior was tested in a two compartment light–dark test. The test apparatus consisted of a illuminated compartment [19 cm (*l*) × 21 cm (*w*) × 20 cm (*d*)] connected with a dark compartment [17 cm (*l*) × 21 cm (*w*) × 20 cm (*d*)] by a 5 cm × 5 cm opening. Mice were placed in the illuminated compartment and allowed to explore the entire apparatus. The total time spent in compartments, distance covered and activity in different compartments, together with the number of transitions between compartments were detected with photo beams (TSE System, Bad Homburg, Germany) for 5 min (Stork et al. 2000).

#### Open field exploration

To further assess the novel environment exploration and anxiety-like behavior in mice, we tested the mice in open field at two consecutive days in an arena measuring 50 cm (*l*) × 50 cm (*w*) with 35 cm high walls for, 20 min each. On day1, testing was done under red light (5 lx low light conditions) and on day2 under bright light (100 lx). Each chamber was divided into corners (12.5 cm × 12.5 cm), rims (25 cm × 12.5 cm) and center (25 cm × 25 cm) regions and exploration was monitored using a video-tracking system (ANY-maze Video tracking system, version 4.50, Stoelting Co, Wood Dale, IL, USA). The distance moved by the mice and percentage of time spent in different regions were measured for 20 min.

#### Spatial discrimination/pattern separation

The task was done as described previously (Bekinschtein et al. 2013) with slight modifications. Briefly, mice were first habituated to the open field arena for two sessions (10 min each) on same day. On the following day, during the sample phase (10 min session), mice encountered identical objects at three locations (A1, A2, A3), with A1 equidistant (24 cm) from both A2 and A3, which were 14 cm apart towards the opposite corner of the arena. During the choice phase 24 h later, two identical objects were presented at the familiar location A1 and the new location A4 in the middle between A2 and A3 (Fig. 4a). Exploration time (expressed as % of time) at different locations for 10 min was recorded and analyzed using ANY-maze and a preference ratio was calculated using the formula: (exploration time at novel location-exploration time at familiar location)/(total time at both locations).

#### Novel object location

This spatial version of the object recognition task was employed in the open field chamber, one day after the exploration in the field during bright light. On the first day of the experiment, mice were habituated to two identical objects (made from Lego blocks, with 0.025 g cumin powder applied as a mild olfactory cue to each object) placed at different locations (each location was assigned to each corner and equidistant (10 cm) from the two walls respective to that corner in an open field arena) B1 and B2. 24 h later, mice encountered one of the objects at novel location B4 (see Fig. 4e). Exploration of objects was monitored for 20 min each using ANY-maze, software and the exploration time (expressed as % of time) at the novel and familiar locations was measured and a preference ratio was calculated as described above.

#### Fear conditioning

WT and cKO mice were tested in a classical auditory cued conditioning paradigm described earlier (Laxmi et al. 2003). The training apparatus (TSE System, Bad Homburg, Germany) comprised of an acrylic glass arena [16 cm (*l*) × 32 cm (*w*) × 20 cm (*d*)] with a grid floor for delivery of electric foot shock. The entire arena was enclosed in a sound-proof cubicle containing speaker, ventilation fan and background noise (70 dB), connected to a computer to measure the different parameters, using photo beam system. Habituation, training and testing sessions were done as described previously (Bergado-Acosta et al. 2008) with minor modifications. Briefly, mice were habituated to the training apparatus with 2 sets of six presentations of a neutral acoustic stimulus (CS− 2.5 kHz, 10 s with 20 s inter stimulus intervals, ISIs) separated by a 2 min pause. During the next day, mice were trained with 2 sets (separated by a 2 min pause) of three presentations of the conditional acoustic stimulus (CS+ 10 kHz, 10 s with 20 s ISIs) each CS+ stimulus terminating with a 1 s unconditional stimulus (US, scrambled foot shock of 0.4 mA). Fear memory towards context and different tones was tested in training context and a neutral context (new standard cage with bedding), respectively, (Online Resource 2b) and freezing behavior (lack of movements except for respiration) was monitored. Fear memory levels were expressed as percentage of time spent freezing during the retrieval sessions.

#### Active avoidance

Mice were tested in a shuttle box (TSE System, Bad Homburg, Germany) as described previously (Sparkman et al. 2005), with minor changes. Briefly, we used a Plexiglas compartment with 36 cm (*l*) × 21 cm (*w*) × 20 cm (*d*) dimensions and with a separator in the middle with 4 cm × 4 cm opening equipped with a grid floor for delivery of foot shocks. Mice were habituated to the apparatus for 3 min on each training day. Conditioned stimuli (CS) (tone: 10 kHz, 65dB) were presented for 20 s in total. After the first 5 s of each tone presentation, an unconditioned stimulus (US) (foot shock: 0.10 mA) was co-presented and foot shock intensity was increased to 0.30 mA if the mice did not shuttle to the other compartment within the 10 s after the presentation of US. The test was performed for 5 consecutive days with 50 trials presented per day and with inter-trial intervals of 20 s. As conditioned responses, crossing to the other chamber with the onset of CS before delivery of the foot shock (0.1 mA) (avoidance response), were measured by photo beam detection (TSE System, Bad Homburg, Germany).

#### Morris water maze

To assess spatial learning and memory, mice were tested in the Morris water maze-submerged platform task in a large round basin (130 cm) containing opaque water (24–26 °C) and a circular platform (10 cm diameter) placed approximately 1.5 cm below the water level. The test was executed as described previously (Montag-Sallaz and Montag 2003) with minor modifications. Mice were subjected to six trials per day for 5 days. They were allowed to swim until they found the platform or until 120 s had elapsed. In the latter case, animals were guided to the platform and allowed to rest for 20 s. The hidden platform remained at a fixed position (South–East) for the first 3 days (18 trials, acquisition phase) and was moved into the opposite quadrant (North–West) for the 2 last days (12 trials, reversal phase). The 1st two trials (trial 19 and 20) on the 1st day of reversal training were considered as probe trials and the time spent in old platform quadrant vs. the new platform quadrant was analyzed to assess the spatial memory. All trials were videotaped and analyzed using the VideoMot 2 system (TSE) and Wintrack open source software (Wolfer and Lipp 1992).

#### Data analysis and statistics

All behavioral data were analyzed and graphs were plotted using MS-Office excel (versions 2010 and 2016) and GraphPad Prism (version 5, USA). For comparison of two groups, data was evaluated by Student’s *t* test (unpaired) or Mann–Whitney *U* test according to the outcome of Shapiro–Wilk normality testing and Chi-square (*χ*^2^) test. Multiple comparisons were done using two-way ANOVA and two-way repeated measures ANOVA, followed by Bonferroni’s posttest. We tested for outliers using the free calculator from Graphpad based on the Grubb’s test (1969) and the Dean and Dixon test (1951). Probability values of < 0.05 were considered as significant.

### Electrophysiology

Adult (3–4 months old) and young (27–33 days old) male *Bsn* cKO mice and their WT littermates were used for electrophysiological experiments (12 h light–dark cycle, lights on at 7 p.m.; room temperature 22 ± 2 °C).

#### Slice preparation

Adult male mice were deeply anesthetized with isoflurane and decapitated. Brains were rapidly (~ 30 to 60 s) removed and placed in cold (4–8 °C) carbogenated (5% CO_2_/95% O_2_) artificial cerebrospinal fluid (aCSF) containing (in mM) 129 NaCl, 21 NaHCO_3_, 3 KCl, 1.6 CaCl_2_, 1.8 MgSO_4_, 1.25 NaH_2_PO_4_ and 10 glucose. Dorsal hippocampal transverse slices (400 µm) were obtained from the septal pole by cutting parasagittal slices at an angle of about 12° using an angled platform (Albrecht et al. 2016). Three to four most dorsal slices were transferred to an interface chamber perfused with aCSF at 34.0 ± 1.0 °C (flow rate: 2.0 ± 0.2 ml/min, pH 7.4, osmolarity ~ 300 mosmol/kg). Slices were incubated for at least 1 h before starting recordings. The experimenter was blind to the genotype of mice.

#### Field potential recordings

Extracellular field recordings were obtained with a glass electrode filled with aCSF (~ 1 MΩ). For DG electrophysiology, the recording electrode was placed at the mid-molecular layer at 70–100 µm depth (Online Resource 5a). Stimulation activating medial fibers in the MPP was performed using a bipolar tungsten wire electrode with exposed tips of ~ 20 µm and tip separations of ~ 75 µm (electrode resistance in aCSF: ~0.1 MΩ). The stimulating electrode was positioned in the middle one-third of the molecular layer, while the recording electrode was placed in the mid-molecular layer as described before (Petersen et al. 2013; Dahl and Sarvey 1989; see Online Resource 5a). The positioning was confirmed by the verification of paired-pulse depression at 50 ms interpulse interval. For CA1 electrophysiology, the recording electrode was placed at the stratum radiatum (SR) of the CA1 subregion, while the stimulation electrode was placed at the proximal CA1 stimulating Schaffer collaterals (SC; Online Resource 5b). Signals were pre-amplified using a custom-made amplifier and low-pass filtered at 3 kHz. Signals were sampled at a frequency of 10 kHz and stored on a computer hard disc for offline analysis.

#### Stimulation protocols

Before obtaining an input–output (I/O) curve, 10–20 min of baseline responses were recorded (0.033 Hz, pulse duration: 100 µs). Once the responses were stabilized, seven intensities ranging from 5 to 50 µA were used to obtain an input/output (I/O) curve. The stimulus intensity that resulted in ~ 40 to 50% of the maximum amplitude was further used for the paired-pulse (PP), long-term potentiation (LTP) and long-term depression (LTD) experiments. PP responses were recorded using intervals from 10 to 500 ms. In the same slice, after recording of a second baseline for 10 min (0.033 Hz), either LTP or LTD was induced. Prior to induction of LTP in the MPP-DG synapse, slices were perfused with 100 µM picrotoxin (PTX) to block GABA_A_ receptor-mediated transmission (Wright and Jackson 2014; Hanse and Gustafsson 1992; Colino and Malenka 1993). To induce LTP at the MPP-DG synapse either one or four trains of high frequency stimuli (HFS; 20 s interval, 100 Hz, 1 s duration) were applied. To induce LTP at the SC-CA1 synapse, two HFS (100 Hz) with 20 s intervals and 1 s duration was applied. At both synapses, LTD was induced by a low frequency simulation (LFS; 900 pulses, 1 Hz) protocol. After LTP or LTD, induction responses were recorded for 40 min (0.033 Hz).

#### Data analysis and statistics

Data were analyzed offline using self-written MATLAB-based analysis tools (MathWorks, Natick, MA, USA). The slopes of fEPSP were analyzed by measuring the slope between 20 and 80% of the maximum fEPSP amplitude (Online Resource 5c). FV volley amplitude was calculated using peak to through amplitudes of descending phase of the FV. fEPSP slope to FV amplitude ratios were obtained over a range of stimulus strengths applied during I/O curves as reported before (Patterson et al. 1996) and statistical analysis was performed using either *t* test (two-tailed) or Mann–Whitney *U* test, after normality test (Shapiro–Wilk Test) and equal variance test were performed. For the analysis of the LTP and LTD, the data were normalized to the average of 10 min baseline before the induction of LTP or LTD. Normalized values obtained from 30 to 40 min after LTP or LTD induction were used to determine genotype differences by *t* test (two-tailed) or Mann–Whitney *U* test, after normality test (Shapiro–Wilk Test) and equal variance test were performed. Statistical comparison of I/O curves and PP responses to determine genotype differences in the adult mice were performed using two-way repeated measures ANOVA followed by posthoc comparison using Fisher LSD Method. Statistical analysis of I/O curves between young and adult mice were performed using two-way repeated measures ANOVA [SigmaPlot for Windows Version 11.0, 2008 (Systat software GmbH, Erkrath)].

## Results

### *Bsn* cKO mice lack Bassoon in excitatory synapses of the hippocampus and cerebral cortex

**Fig. 1:**
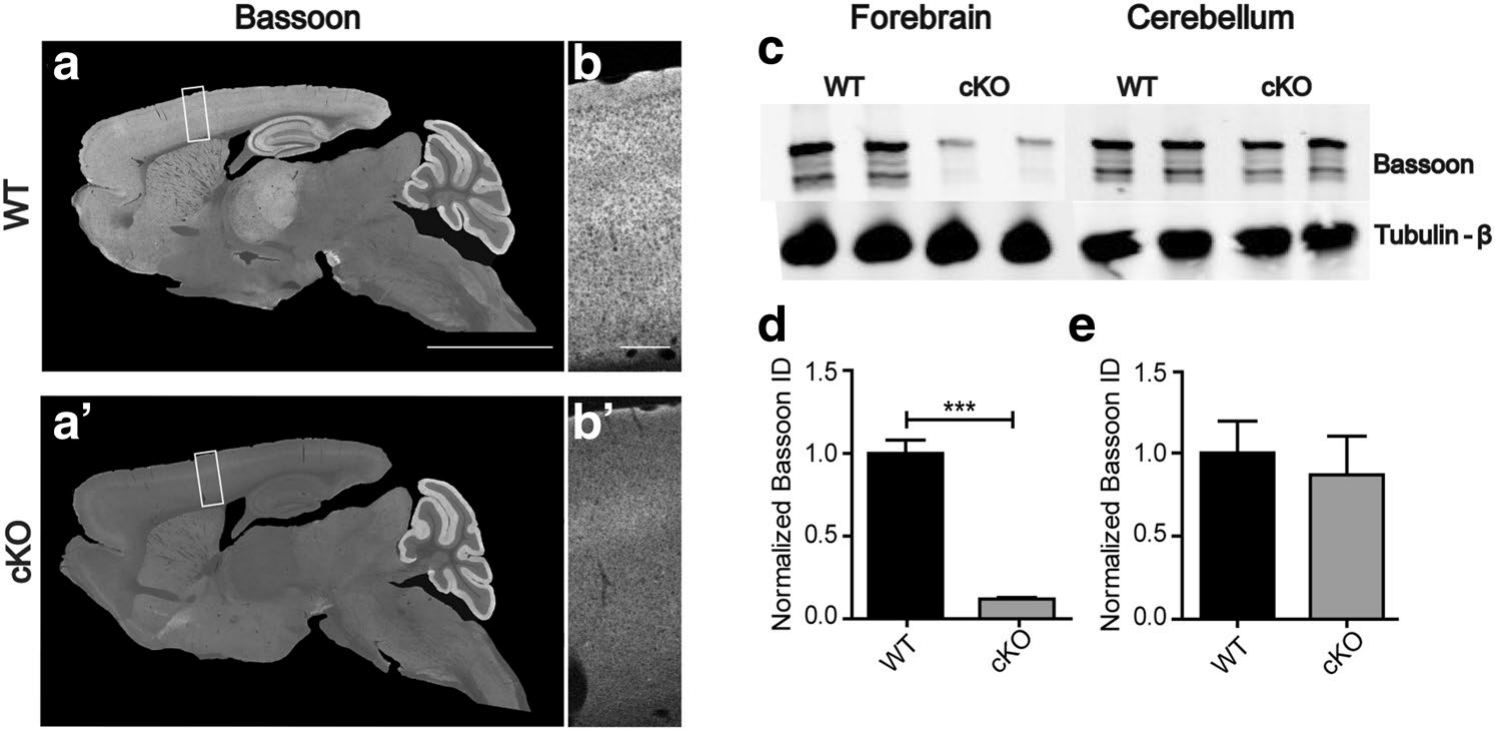
*Bsn* cKO mice lack Bassoon in forebrain region. Overview of sagittal brain sections from wild-type (WT) (**a**) and littermate *Bsn* cKO (**a′**) mice stained for Bassoon. Clear reduction in the expression of Bassoon is evident in the cKO forebrain regions (cerebral cortex, hippocampus); whereas cerebellum, midbrain and brain stem from both genotypes show no difference in Bassoon expression. High magnification details display Bassoon expression in the cerebral cortex of WT (**b**) and *Bsn* cKO mice (**b**′). **c** Western blots (10 µg protein/lane) of forebrain and cerebellum homogenates from WT and cKO brains showing expression levels of Bassoon and β-tubulin (loading control). **d** A strong reduction of Bassoon expression (~ 15% of WT levels) is evident in the forebrain of cKO mice (integrated density values normalized to WT). **e** No significant change in Bassoon expression can be observed in the cerebellum (*N* = 5). Scale bars 3 mm in **a**, 250 µm in **b**. All values are mean ± SEM; ***p ≤ 0.001, Student’s *t* test

**Fig. 2:**
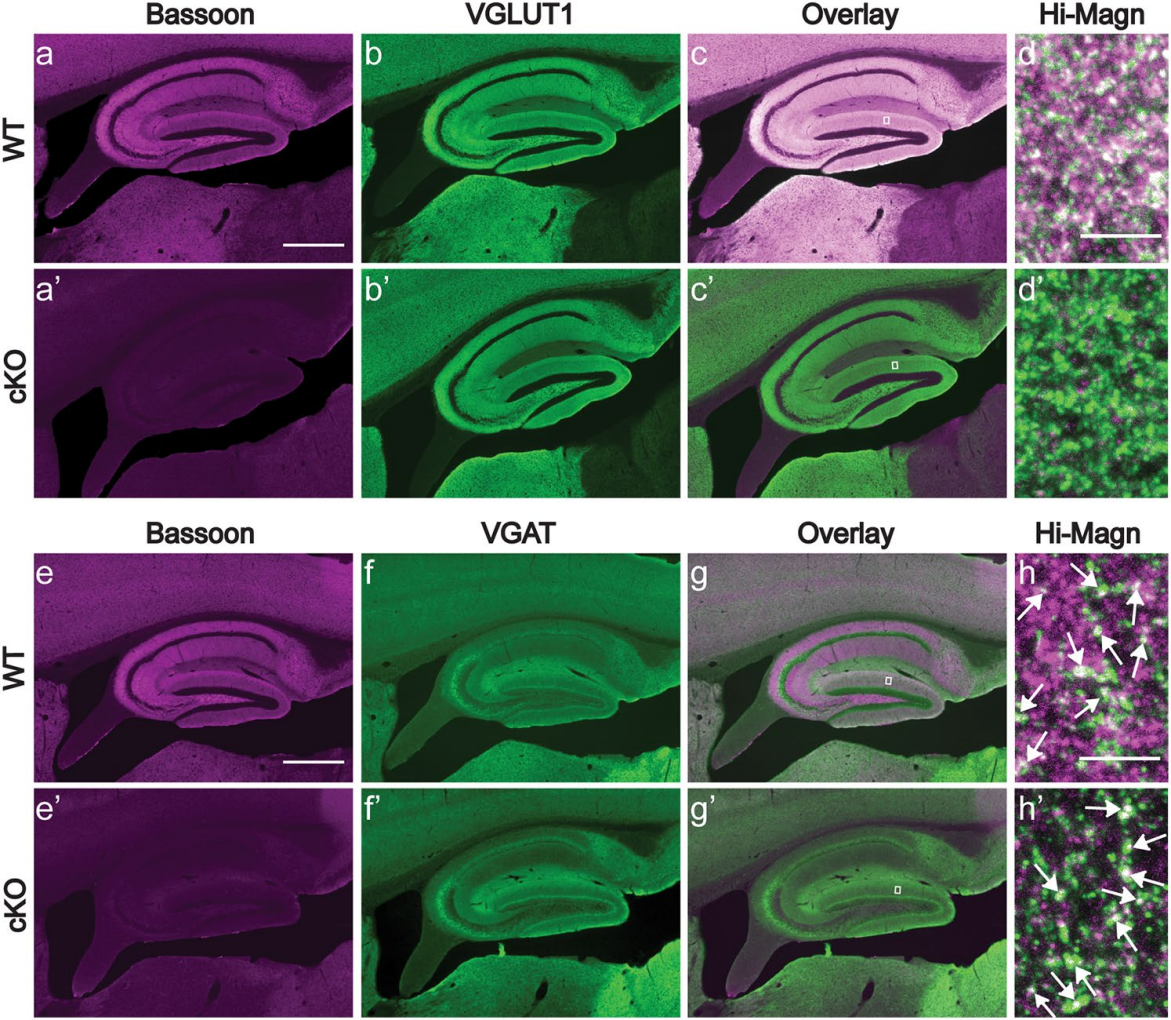
*Bsn* cKO mice lack Bassoon in excitatory synapses of the hippocampus. Immunoreactivity of Bassoon (magenta, **a, a′**), VGLUT1 (green, **b, b′**) antibodies in hippocampal sections from WT and cKO mice depict the localization of Bassoon in all neuropil layers in WT (**c, d**) and loss of this immunoreactivity in cKO mice (**c′, d′**). For comparison, immunoreactivity of Bassoon (magenta, **e, e′**) and VGAT (green, **f, f′**) antibodies shown in the hippocampus of both WT (**g**) and cKO mice (**g′**) shows a more disparate distribution. Entangled areas from overlay images (high magnification images from molecular layer of DG, **h** and **h′**) demonstrate that Bassoon is still expressed in inhibitory synapses (indicated by white arrows). Residual labeling is evident in some non-GABAergic synapses, potentially arising from neuromodulatory afferences. *Hi-Magn* high magnification. Scale bar in **a, e** is 500 µm and high magnification **d, h** is 5 µm

Bassoon is present at virtually all excitatory and inhibitory synapses throughout the brain (Richter et al. 1999). To study Bassoon function specifically in glutamatergic neurons of the forebrain, we generated mice in which exon 2 of the *Bsn* gene was flanked by loxP sites (*Bsn2*^*lx*/*lx*^; see Online Resource 1a–d) and bred them with mice expressing Cre recombinase under the control of the *empty spiracle homeobox-1* (*Emx1*) promoter (knock-in *Emx1*^*Cre*/+^), which drives recombination in forebrain excitatory neurons and astroglia, but not in GABAergic interneurons. To verify the selective depletion of Bassoon from glutamatergic forebrain synapses in the resulting *Bsn2*^*lx*/*lx*^*Emx1*^*Cre*/+^ (in short: *Bsn* cKO) mice, sagittal brain sections were stained with Bassoon antibody. Littermates (*Bsn2*^*lx*/*lx*^*Emx1*^+/+^ or in short WT mice) served as controls as they expressed wild-type levels of Bassoon. As expected, when compared to the WT mice (Fig. 1a,b), a strongly reduced staining intensity was observed in the cerebral cortex and hippocampus of *Bsn* cKO mice (Fig. 1a′, b′). In the cerebellum and other regions like midbrain, brainstem, by contrast no difference was evident in Bassoon expression between the genotypes (Fig. 1a, a′). Next, immunoblot analysis was performed to quantify the expression levels of Bassoon protein in forebrain (cerebral cortex and hippocampus) and cerebellar homogenates (Fig. 1c). Bassoon expression was reduced by 85% in forebrain homogenate of *Bsn* cKO mice when compared to WT mice (Fig. 1d, *t*(8) = 10.74, *p* < 0.0001, Student’s *t* test), whereas Bassoon levels were unchanged in cerebellar homogenates (Fig. 1e, *t*(8) = 0.4317, *p* = 0.6773). Finally, we assessed the Bassoon depletion in hippocampal sections, and examined its synaptic specificity. Sections were double stained with antibodies against Bassoon and VGLUT1 as a marker for glutamatergic presynapses (Fig. 2a–d, a′–d′) or VGAT, a marker for inhibitory presynapses (Fig. 2e–h, e′–h′). High-resolution confocal images of the hippocampus show a profound co-localization of Bassoon and VGLUT1 in WT (Fig. 2d) but not in *Bsn* cKO mice (Fig. 2d′), while persistently high co-localization of Bassoon and VGAT was observed in *Bsn* cKO mice (Fig. 2h′). Accordingly, it is evident that ablation of Bassoon occurs at forebrain excitatory but not at inhibitory synapses in *Bsn* cKO mice.

### Behavioral assessment of *Bsn* cKO mice

**Fig. 3:**
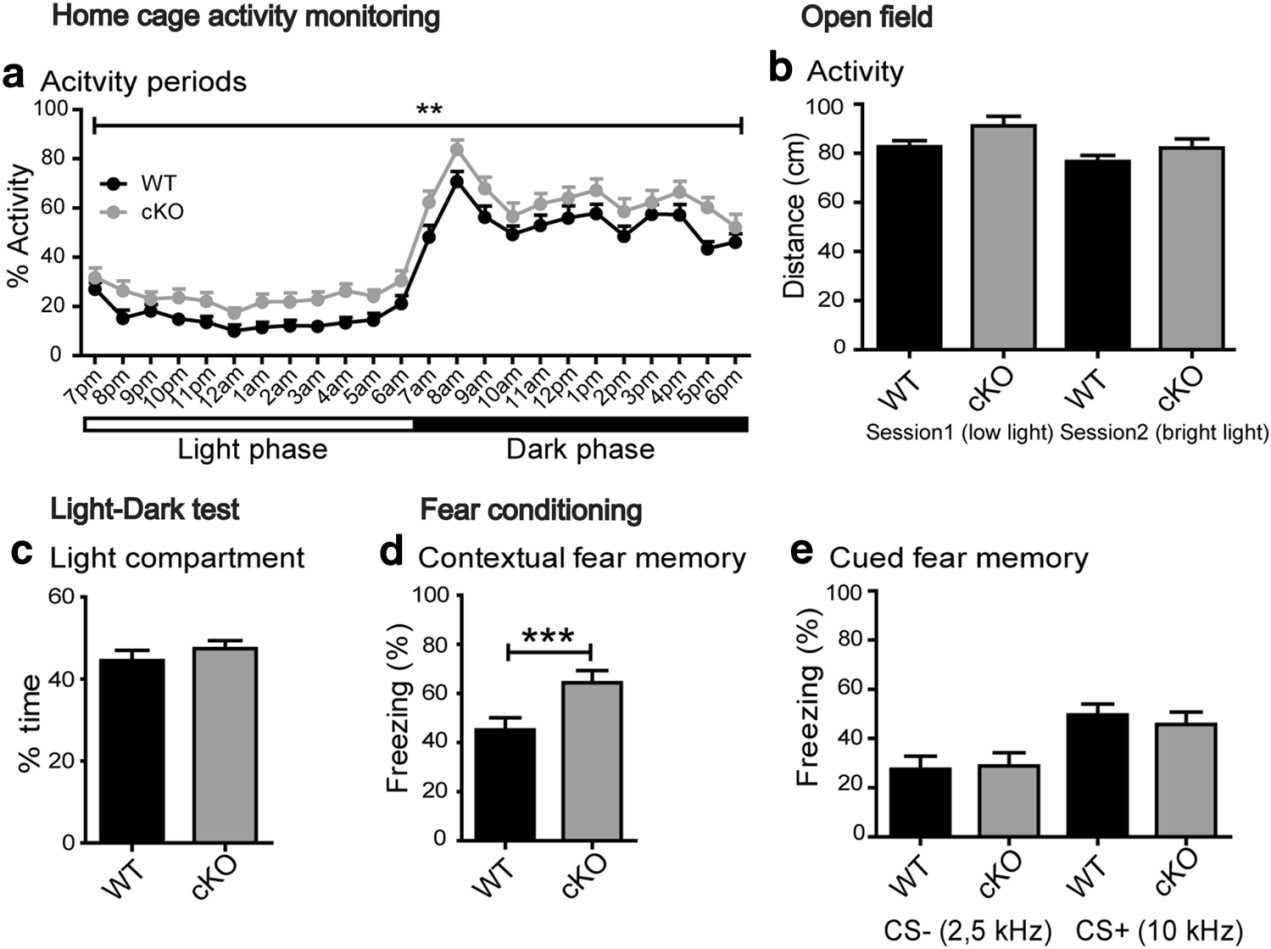
*Bsn* cKO mice display increased contextual fear memory. **a** Analysis of home cage activity in WT (*N* = 20) and Bsn cKO (*N* = 19) mice suggests normal circadian rhythm with increased locomotion during the dark phase. Activity values of cKO mice are generally increased as compared to WT mice. **b** In an open field test WT and cKO mice show similar levels of exploratory activity under both low light and bright light illumination (distance explored) (WT: *N* = 24; cKO: *N* = 19). **C** Anxiety levels are unchanged in cKO mice as indicated by the percentage of time spent in light chamber during the light–dark test (WT: *N* = 12; cKO: *N* = 10). **D** Increased freezing towards the shock context is observed in cKO mice indicating an enhanced contextual fear memory. **e** By contrast no genotype difference is evident in the conditioned fear response towards the auditory tones in neutral context; both groups furthermore clearly differentiate the neutral acoustic stimulus (CS−) and conditioned acoustic stimulus (CS+) (WT: *N* = 13; cKO: *N* = 11). All values are mean ± SEM; **p ≤ 0.01, ***p ≤ 0.001, two-way repeated measures ANOVA with Bonferroni post hoc test (**a**–**e**), Mann Whitney *U* test (**c**)

Male *Bsn* cKO mice and WT littermates were analyzed in different behavior paradigms (Table 1). When analyzing home cage activity, a significant effect of the day time was observed with strongly increased activity during the 12 h dark phase of the diurnal cycle (Fig. 3a, *F*(23,851) = 95.97, *p* < 0.0001, two-way repeated measures ANOVA). A significant genotype effect [*F*(1,37) = 9.88, *p* = 0.0033] that did not show an interaction with day time [*F*(23,851) = 0.49, *p* = 0.9795] revealed that home cage activity was increased in *Bsn* cKO as compared to WT mice during both dark and light phases.

**Table 1:** Overview of the behavioral assessment of *Bsn* cKO mice and their WT littermates

Behavioral domain	Test	Behavior of *Bsn* cKO mice as compared to WT
Activity and anxiety	Home cage activity	Hyperactive (Fig. 3a)
	Open field	No difference (Fig. 3b, and Online Resource 2a)
	Light–dark test	No difference (Fig. 3c)
Novelty recognition and spatial learning	Novel object location	Non-significant trend towards novelty preference (Fig. 4e–h)
	Spatial discrimination/pattern separation	Increased preference for the novel location (Fig. 4a–d)
	Morris water maze	No difference (Online Resource 4)
Fear learning	Contextual fear memory	Enhanced (Fig. 3d and Online Resource 2c)
	Cue fear memory	No difference (Fig. 3e and Online Resource 2d)
	Active avoidance	No difference (Online Resource 2e)

**Fig. 4:**
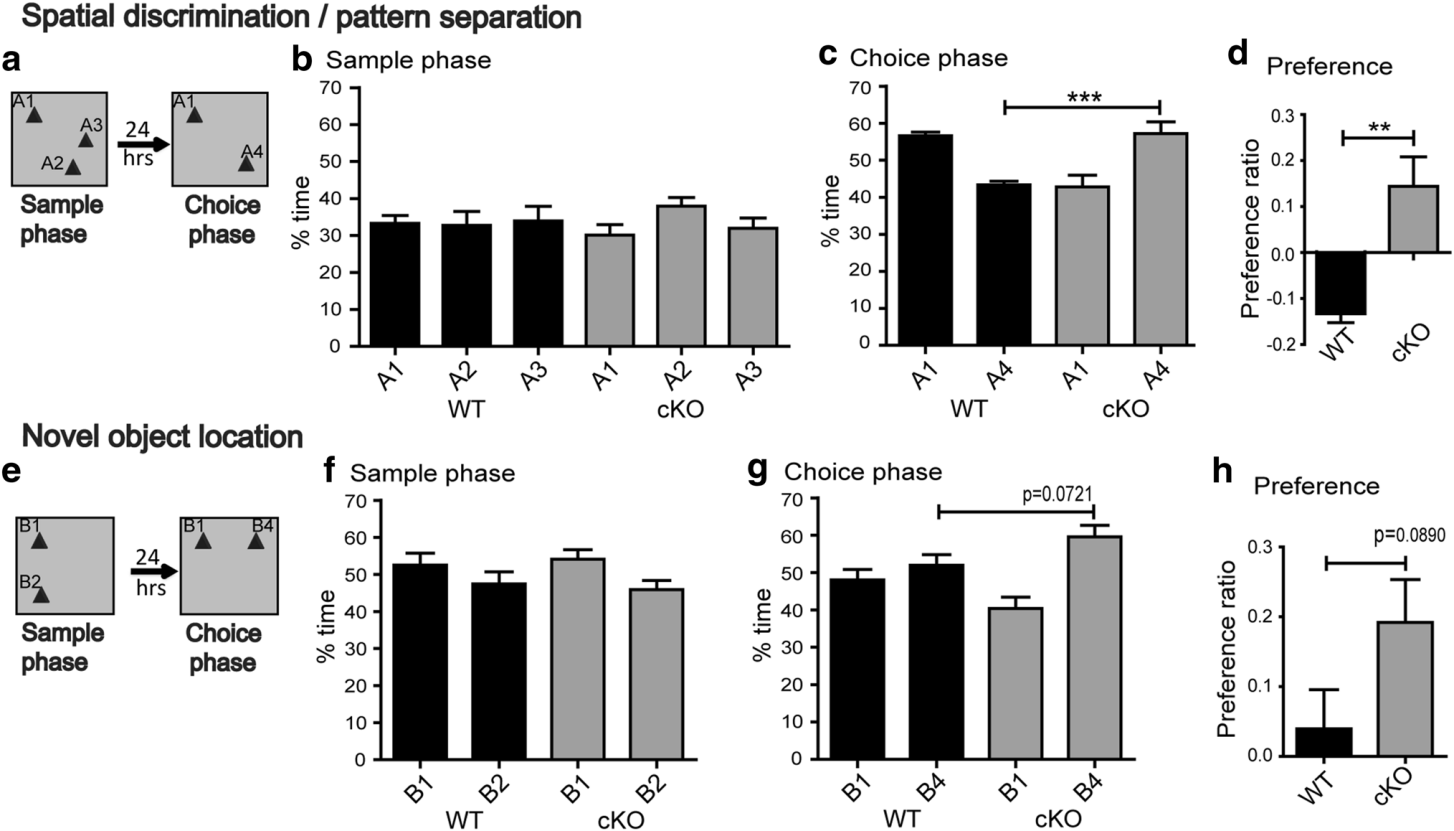
*Bsn* cKO mice display increased preference for the novel location in a spatial discrimination/pattern separation task. **a** Schematic illustration of the spatial discrimination/pattern separation task. **b** Both genotypes show a similar exploration of all three test objects during the sample phase (memory acquisition). **c** During the choice phase (memory test) WT mice spend more time exploring the object at the familiar location than the object at the novel location. By contrast, cKO mice clearly prefer the object at the novel location. **d** Preference ratio analysis reveal the increased preferences of the cKO mice for the object in the novel location during the choice phase (WT: *N* = 7; cKO: *N* = 9). **e** Schematic illustration of the novel object location task. **f** Both groups equally explore the objects in both the locations during the sample phase. **g** During the choice phase, cKO mice explore the object at the novel location much more than the object at the familiar location, whereas WT mice show only a weak preference. **h** Preference ratio reveal a non-significant trend towards an increased preference of cKO during the choice phase (WT: *N* = 8; cKO: *N* = 7). All values are mean ± SEM; **p ≤ 0.01, two-way ANOVA with Bonferroni post hoc test (**b, f**), Student’s *t* test and Mann Whitney *U* test (**c, d, g, h**)

Exploratory activity in an open field was tested during the dark phase of the cycle using low light and bright light test sessions. Two-way repeated measures ANOVA revealed an effect of the test session [*F*(1,41) = 15.78, *p* = 0.0003] but no effect of genotype [*F*(1,41) = 3.22, *p* = 0.0803] or genotype × session interaction [*F*(1,41) = 0.64, *p* = 0.4276] concerning the distance travelled in the open field (Fig. 3b). Furthermore, we compared the time mice spent exploring the center of the open field arena between the genotypes as a measure of anxiety-related behavior. Again, there was an effect of the test session [*F*(1,41) = 15.01, *p* = 0.0004], but no genotype effect [Online Resource 2a; *F*(1,41) = 0.07, *p* = 0.7940] or genotype × session interaction [*F*(1,41) = 1.04, *p* = 0.3137] was observed.

Similarly, in the light–dark test for anxiety-like behavior, *Bsn* cKO and WT mice spent comparable time periods in the illuminated compartment (Fig. 3c; WT: 44.54 ± 2.49%; *Bsn* cKO: 47.41 ± 1.98; *U* = 53.50, *p* = 0.6923, Mann–Whitney *U* test). The distance (expressed as % of total) travelled in the illuminated compartment (WT: 39.38 ± 1.62%; *Bsn* cKO: 42.14 ± 1.32%; *U* = 42, *p* = 0.2485) as well as the number of transitions between illuminated and dark compartments were not different between the genotypes (WT: 6.75 ± 0.77; cKO: 6.40 ± 0.54; *U* = 50.50, *p* = 0.5469).

### *Bsn* cKO mice exhibit altered contextual fear conditioning

Bsn cKO mice and WT littermates were tested in a combined contextual and cued fear conditioning paradigm (Online Resource 2b). Bsn cKO mice displayed significantly altered fear response in contextual fear conditioning (Fig. 3d and Online Resource 2c). Freezing analysis using two-way repeated measures ANOVA, revealed increased freezing levels in both genotypes during contextual retrieval when compared to baseline freezing levels and freezing levels prior to training session [effect of test session *F*(2,44) = 191.01, *p* < 0.0001]. This analysis further indicated a significant effect of genotype [*F*(1,22) = 9.47, *p* = 0.0055] and genotype × session interaction [*F*(2,44) = 5.36, *p* = 0.0083]. Post hoc comparison of genotypes revealed a significant difference between genotypes during contextual retrieval (Fig. 3d, *p* < 0.001, Bonferroni test), but not in baseline freezing levels (WT: 1.73 ± 0.33%; *Bsn* cKO: 2.42 ± 1.69%; *p* > 0.05) or during pre-training (WT: 4.94 ± 0.76%; *Bsn* cKO: 9.70 ± 2.79%; *p* > 0.05). On the following day, mice were tested for their memory towards the auditory cue presented in a neutral context (Fig. 3e and Online Resource 2d). Both genotypes showed low freezing levels to the neutral context alone or to the non-conditioned auditory test stimulus (CS−), but strong freezing to the CS+ [effect of test stimulus: *F*(1,22) = 17.09, *p* = 0.0004, two-way repeated measures ANOVA]; however, no evidence was found for a genotype effect [*F*(1,22) = 0.06, *p* = 0.8123] or a genotype × test stimulus interaction [*F*(1,22) = 0.32, *p* = 0.5794].

To control for a bias due to potential differences in pain sensitivity between genotypes, mice were exposed to a series of foot shocks increasing from 0.1, 0.2 and 0.3 mA. Neither the freezing response nor flinching behavior nor vocalization was different between genotypes (Online Resource 2f, g). Furthermore, to control for genetic background, Cre expression and Emx1 haplodeficiency, we also tested Emx1^+/+^ (without Cre) and Emx1^Cre/+^ mice in the behavioral paradigms used for the characterization of *Bsn* cKO mice. None of the analyzed parameters was different between the two genotypes (Online Resource 3) confirming that the driver line alone has no phenotype by itself in this paradigm.

### Normal active avoidance learning in *Bsn* cKO mice

Since *Bsn*^*ΔEx4*/*5*^ mice were shown to display increased performance in an active avoidance task (Ghiglieri et al. 2010), we also tested *Bsn* cKO mice in this paradigm. However, both the *Bsn* cKO and WT mice learned the task efficiently showing an increase of avoidance reactions over time [*F*(4,72) = 39.06, *p* < 0.0001, two-way repeated measures ANOVA], and no genotype differences [*F*(1,18) = 0.13, *p* = 0.7226] or session × genotype interactions [*F*(4,72) = 1.06, *p* = 0.3813] were apparent (Online Resource 2e).

### *Bsn* cKO mice display increased preference for the novel location in a spatial discrimination/pattern separation task

To further test the hippocampal functions of *Bsn* cKO mice, we analyzed their behavior in a spatial discrimination/pattern separation task and a novel object location task. Both tasks are based on rodents’ innate behavior to preferentially explore new spatial settings (Ennaceur and Delacour 1988). In the spatial discrimination/pattern separation task (Fig. 4a), both *Bsn* cKO and WT mice explored all three locations equally well during sample phase [Fig. 4b, *F*(2,42) = 0.78, *p* = 0.4669, two-way ANOVA] with no main effect of genotype [*F*(1,42) = 0.00, *p* = 1.000] genotype × location interaction [*F*(2,42) = 1.15, *p* = 0.3263], indicating no location bias. During the choice phase, genotype comparisons revealed a significant difference between the WT and cKO mice at the novel locations (Fig. 4c; A4—WT: 43.37 ± 0.98%; *Bsn* cKO: 57.20 ± 3.21; *p* = 0.0002, Mann–Whitney *U* test). We also calculated preference ratios to illustrate the relative preference for either location; these differed significantly between WT and *Bsn* cKO mice [Fig. 4d, *t*(14) = 3.670, *p* = 0.0025, Student’s *t* test]. One WT was identified as an outlier based on the Grubb’s test (1969) and the Dean and Dixon test (1951) and excluded from the analysis.

In the novel object location task, during sample phase, WT and *Bsn* cKO mice were habituated to two identical objects in different locations (Fig. 4e) and significant effect of location [Fig. 4f, *F*(1,26) = 5.04, *p* = 0.0335, two-way ANOVA] with no main effect of genotype [*F*(1,26) = 0.00, *p* = 1.0000] and location × genotype interaction [*F*(1,26) = 0.29, *p* = 0.5966] was observed. During the choice phase 24 h later, comparison of genotypes at novel location revealed no significant changes between the WT and cKO mice (Fig. 4g; B4—WT: 51.97 ± 2.82%; *Bsn* cKO: 59.60 ± 3.06; *p* = 0.0721, Mann–Whitney *U* test). We also found a comparable non-significant trend for an increased novel location preference ratio in *Bsn* cKO mice, when compared to WT mice [Fig. 4h, *t*(13) = 1.838, *p* = 0.0890, Student’s *t* test].

### Unaltered performance of *Bsn* cKO mice in Morris water maze

We further examined *Bsn* cKO and WT mice in the Morris water maze (Online resource 4). During the acquisition phase, a significant learning rate was observed over the three training days with gradually decreasing escape latency to find the platform [effect of training day: *F*(2,32) = 7.32, *p* = 0.0024, two-way repeated measures ANOVA] and reduction of path length [effect of training day: *F*(2,32) = 17.55, *p* < 0.0001] in both the groups (Online resource 4a, b). Main effects of genotype were observed neither for the escape latency [*F*(1,16) = 2.55, *p* = 0.1297] nor for the total path length [*F*(1,16) = 1.94, *p* = 0.1832] and no genotype × training day interaction was observed neither for escape latency [*F*(2,32 = 0.05, *p* = 0.9477] nor for path length [*F*(2,32) = 0.13, *p* = 0.8751]. During the probe trials, both the WT and *Bsn* cKO mice spent comparable time (%) in the old platform quadrant [Probe trial1: *t*(16) = 0.4694, *p* = 0.6451; Probe trial2: *t*(16) = 0.8809, *p* = 0.3914, Student’s *t* test] when compared to new platform quadrant [Probe trial1: *t*(16) = 0.4289, *p* = 0.6737; Probe trial2: *t*(16) = 0.3163, *p* = 0.7559] (Online resource 4c and d). During reversal training, with the platform shifted to a new position, no significant effect of genotype in escape latency [*F*(1,16) = 1.68, *p* = 0.2130] and path length [*F*(1,16) = 0.93, *p* = 0.3492] or genotype × training day interaction effect in escape latency [*F*(1,16) = 2.69, *p* = 0.1202] and path length [*F*(1,16) = 2.95, *p* = 0.1054] were found.

### Increased excitability and baseline synaptic transmission in the dorsal dentate gyrus of *Bsn* cKO mice

**Fig. 5:**
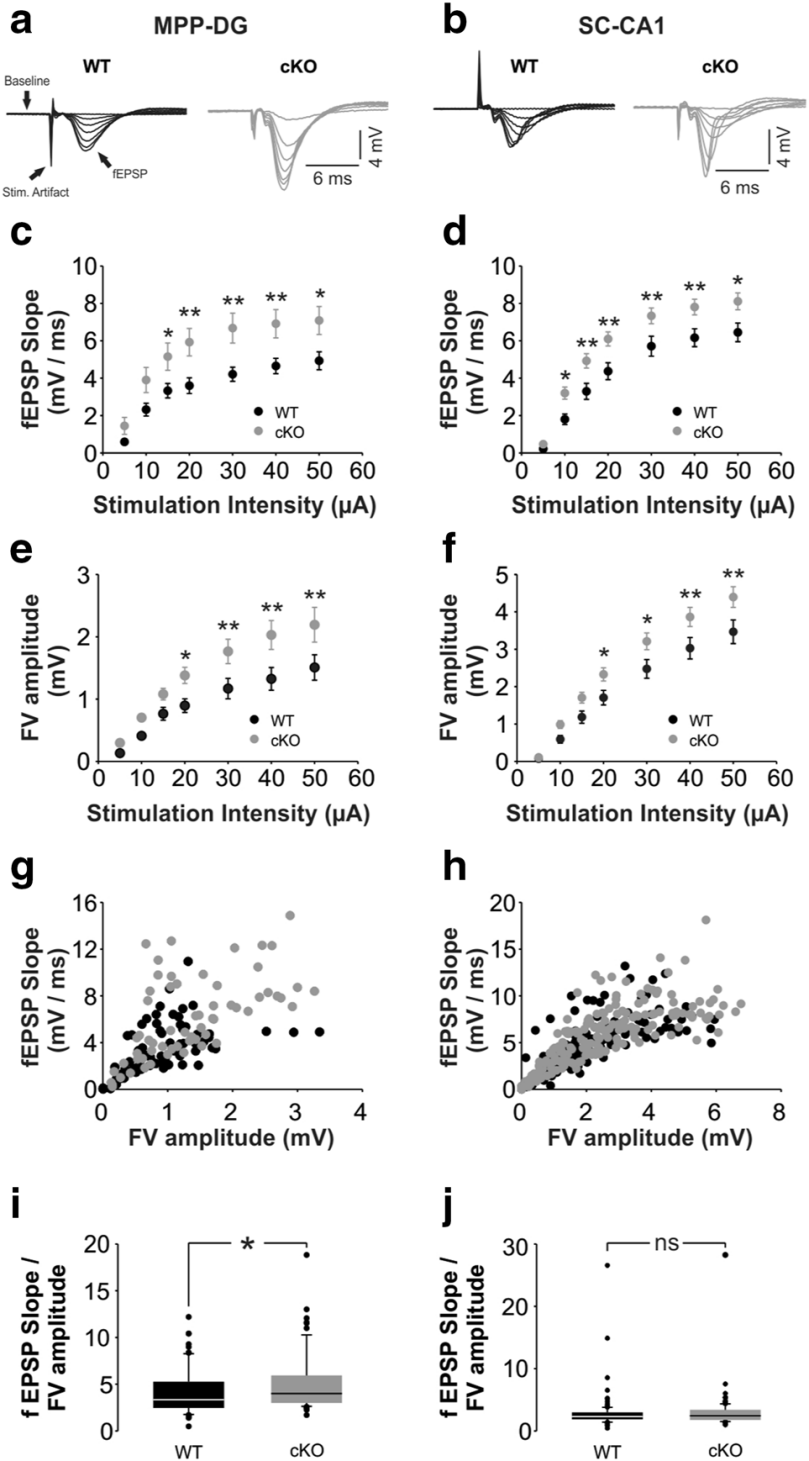
Increased baseline transmission in *Bsn* cKO mice. **a,b** Merged traces of field excitatory postsynaptic potential (fEPSP) responses to increasing stimulation strengths (5–50 µA) at the MPP-DG (**a**) or SC-CA1 (**b**) synapse (WT: black; *Bsn* cKO: gray). Baseline recordings (5 ms) before each stimulation, the stimulation artifacts and fEPSPs are indicated by the arrows. Note the augmented fEPSPs in the cKO mice. **c, d** Summary graphs indicating increased synaptic excitability in both MPP-PP (**c**) and SC-CA1 (**d**) synapses of *Bsn* cKO mice (DG: *N* = 5 mice, *n* = 22 slices; CA1: *N* = 7 mice, *n* = 34 slices) compared to WT mice (DG: *N* = 6 mice, *n* = 21 slices; CA1: *N* = 5 mice, *n* = 27 slices). **e, f** Summary graphs indicating increased presynaptic fiber volley (FV) amplitude in both MPP-PP (**e**) and SC-CA1 (**f**) synapses of *Bsn* cKO mice (DG: *N* = 5 mice, *n* = 9 slices; CA1: *N* = 7 mice, *n* = 29 slices) compared to WT mice (DG: *N* = 6 mice, *n* = 11 slices; CA1: *N* = 5 mice, *n* = 23 slices). **g, h** Single FV amplitudes (mV) plotted against fEPSP slopes (mV/ms) for MPP-DG (**g**) and SC-CA1 (**h**) pathway. Note the specific increase in fEPSP slope values in response to similar preysnaptic FV amplitudes in the MPP-DG synapse of cKO mice. **i, j** Graphs summarizing fEPSP slope to FV amplitude ratios for MPP-DG (**i**) and SC-CA1 (**j**) pathway. Note the specific increase in ratios in the MPP-SC indicating an increased baseline synaptic efficacy. All values are expressed as mean ± SEM. *Significant difference to WT with *p* ≤ 0.05; **p ≤ 0.01; *ns* not significant (Two-way repeated ANOVA followed by posthoc comparison using Fisher LSD Method). *MPP* medial perforant path, *DG* dentate gyrus, *SC* Schaffer collaterals, *CA1* Cornu ammonis area 1

Next, we investigated electrophysiological properties of hippocampal synapses of *Bsn* cKO mice in parasagittal brain slices including the dorsal hippocampus. To determine the baseline excitability and synaptic efficacy in MPP-DG and Schaffer collateral (SC)-CA1 synapses, we measured the fEPSP slopes and fiber volley (FV) amplitudes obtained from input–output (I–O) curves (Online Resource 5). At both MPP-DG and SC-CA1 synapses, we observed a significant increase not only in the fEPSP slopes [Fig. 5a–d; MPP-DG: *F*(1,41) = 6.063, *p* = 0.018; SC-CA1: *F*(1,56) = 7.614, *p* = 0.008, two-way repeated measures ANOVA], but also in the FV amplitudes [Fig. 5e, f; MPP-DG: *F*(1,18) = 5.598, *p* = 0.029; SC-CA1: *F*(1,48) = 5.606, *p* = 0.022, two-way repeated measures ANOVA] in *Bsn* cKO vs. WT slices. To elucidate whether the baseline synaptic efficacy is also augmented in *Bsn* cKO mice, fEPSP slope to FV amplitude ratios were calculated. Indeed, in MPP-DG synapses, the fEPSP-to-FV amplitude ratio is significantly increased indicating an augmented baseline transmission (Fig. 5g, i; WT: 4090.4 ± 289.6, *Bsn* cKO: 5139.2 ± 404.7; *U* = 1739.0, *p* = 0.02, Mann–Whitney *U* test). However, there was no significant alteration in the fEPSP slope to FV amplitude ratio in the SC-CA1 pathway (Fig. 5h, j; WT: 2748.5 ± 221.5, *Bsn* cKO: 2668.1 ± 84.2; *U* = 11051.0, *p* = 0.304).

### Unaltered short and long-term plasticity in the dorsal hippocampus of *Bsn* cKO mice

**Fig. 6:**
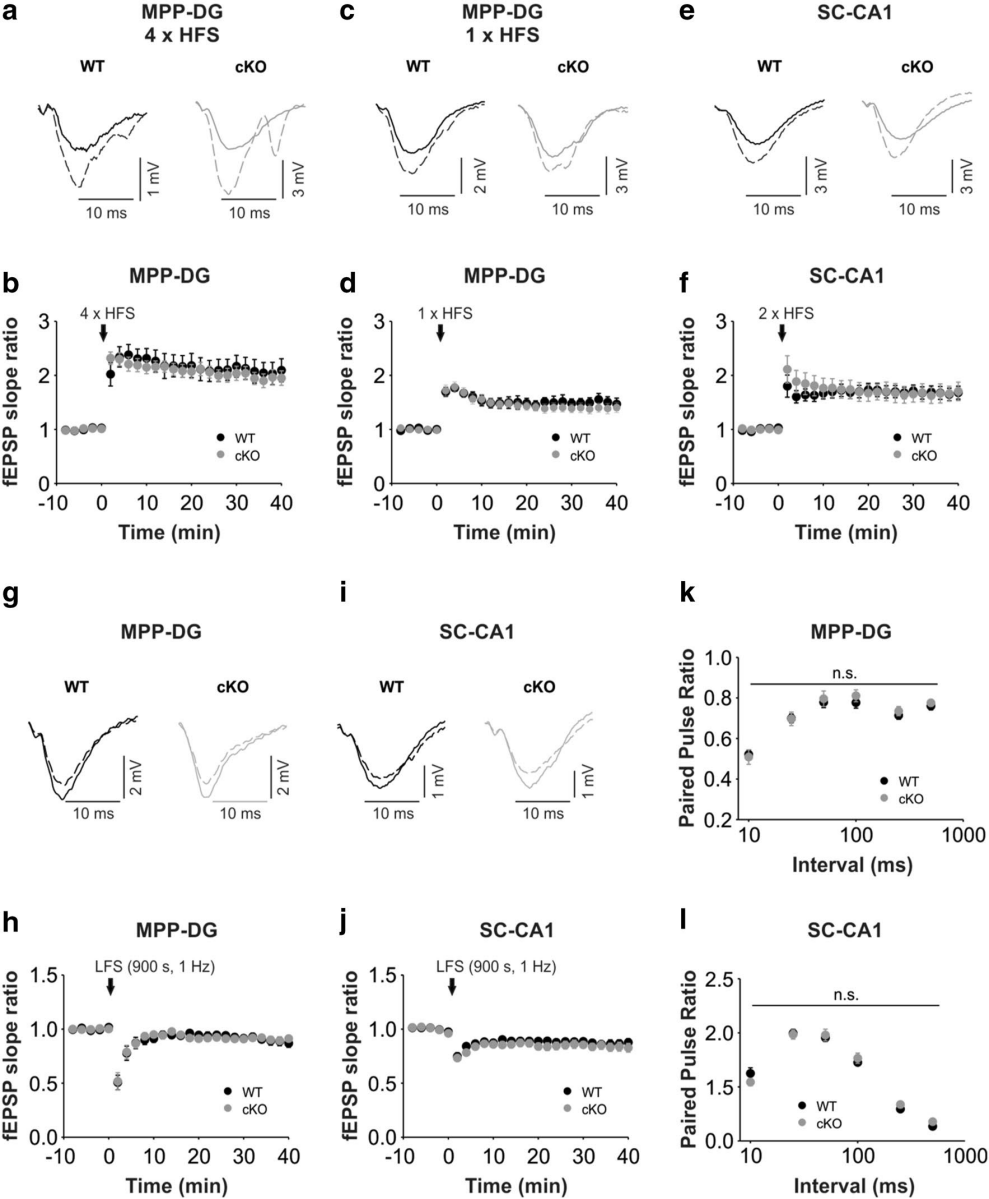
Unaltered long- and short-term plasticity in the dorsal hippocampus of *Bsn* cKO mice. **a, c** Representative control fEPSP traces (WT: black line; *Bsn* cKO: gray line) and traces 35–40 min after (dashed lines) strong (4 × HFS, 1 s, 100 Hz; WT: *N* = 5 mice, *n* = 9 slices, cKO: *N* = 4 mice, *n* = 10 slices) (**a**) weak (1 × HFS, 1 s, 100 Hz; WT: *N* = 3 mice, *n* = 5 slices, cKO: *N* = 4 mice, *n* = 7 slices) (**c**) LTP induction protocols in the MPP-DG pathway. **b, d** Summary graphs showing similar LTP in both genotypes after (**b**) strong and (**d**) weak LTP induction. **e** Representative control fEPSP traces (WT: black line; *Bsn* cKO: gray line) and traces 35–40 min after (dashed lines) LTP induction (2 × HFS, 1 s, 100 Hz; WT: *N* = 3 mice, *n* = 8 slices, cKO: *N* = 3 mice, *n* = 10 slices) in the SC-CA1 pathway. **f** Summary graph showing comparable LTP in both genotypes in the SC-CA1 pathway. **g, i** Representative control fEPSP traces (WT: black line; *Bsn* cKO: gray line) and traces 35–40 min after (dashed lines) LTD induction (LFS, 900 s, 1 Hz) in the MPP-DG pathway (**g;** WT: *N* = 4 mice, *n* = 10 slices, cKO: *N* = 4 mice, *n* = 8 slices) and SC-CA1 (**i** WT: *N* = 4 mice, *n* = 7 slices, cKO: *N* = 4 mice, *n* = 7 slices) pathways. **h, j** Summary graphs showing no statistical difference between genotypes in the MPP-DG and SC-CA1 pathways. **k, l** Summary graphs indicating no significant difference in paired-pulse ratios at interval 10–500 ms between genotypes in the MPP-DG (**k** WT: *N* = 6 mice, *n* = 19 slices, cKO: *N* = 5 mice, *n* = 20 slices) and SC-CA1 synapses (**l** WT: *N* = 5 mice, *n* = 27 slices, cKO: *N* = 7 mice, *n* = 35 slices). All values are expressed as mean ± SEM. ns = not significant (**a**–**j** Student’s *t* test or Mann Whitney *U* test; **k, l** Two-way repeated ANOVA followed by post hoc comparison using Fisher LSD Method)

Augmented baseline excitability in *Bsn* cKO mice might result in altered synaptic plasticity. Thus, we determined the short- and long-term plasticity in both MPP-DG and SC-CA1 synapses. However, no significant alteration in the ability to produce LTP in the MPP-DG synapses was observed using both strong (Fig. 6a, b; *t*(17) = 0.628; WT: 200.1 ± 20.6%, *Bsn* cKO: 195.9 ± 12.5%; *p* = 0.628, Student’s *t* test) and weak (Fig. 6c, d; WT: 150.1 ± 9.7%, *Bsn* cKO: 139.3 ± 8.8%; *U* = 11.0, *p* = 0.343, Mann–Whitney *U* test) stimulation protocols. Similarly, in the SC-CA1 pathway, the level of potentiation was comparable between the two genotypes (Fig. 6e, f; WT: 167.3 ± 11.5%, *Bsn* cKO: 166.6 ± 15.9%; *U* = 35.0, *p* = 0.689). Furthermore, the level of LTD produced by standard LFS protocol was similar between the genotypes in both synapses [Fig. 6g, h; MPP-DG: WT: 90.9 ± 1.9%, *Bsn* cKO: 89.4 ± 33.9%; *U* = 38.0, *p* = 0.894; Fig. 6i, j. SC-CA1: WT: 86.8 ± 3.3%, *Bsn* cKO: 83.7 ± 3.8%; *t*(12) = 0.615, *p* = 0.550]. Finally, to elucidate a potential alteration in short-term plasticity, we measured paired-pulse responses using intervals ranging from 10 to 500 ms. In both synapses, there were no significant alterations at any interval [Fig. 6k, l; MPP-DG: *F*(1, 37) = 0.147, *p* = 0.703; SC-CA1: *F*(1, 60) = 0.064, *p* = 0.802, two-way repeated measures ANOVA].

### *Bsn* cKO mice display morphological changes of dentate gyrus granule cells and a moderately increased brain volume

**Fig. 7:**
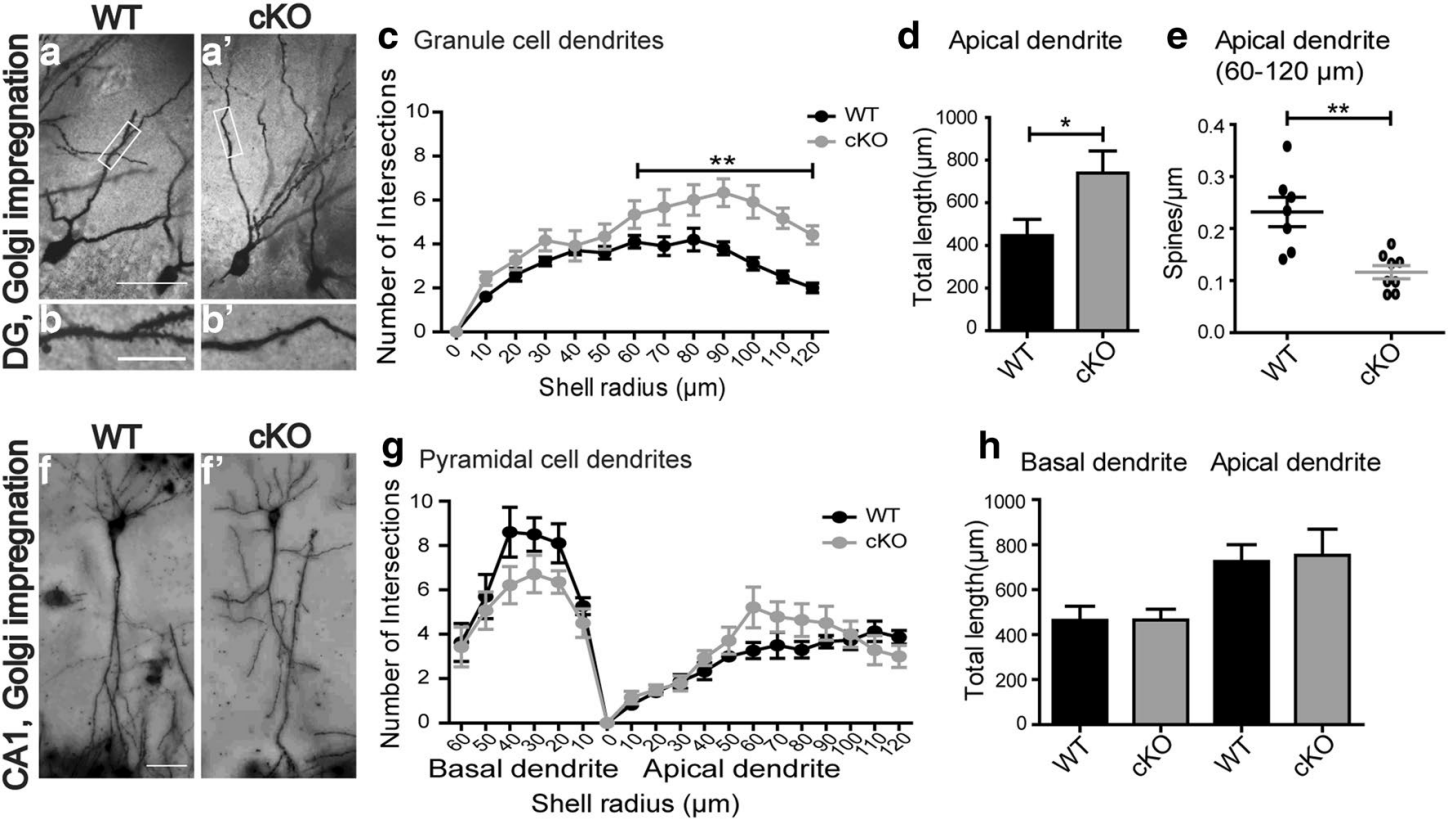
Increased dendritic complexity, increased length and reduced spine density of DG granule cells in *Bsn* cKO mice. Example images of Golgi impregnation of dentate gyrus (DG) granule cells from WT (**a**) and *Bsn* cKO mice (**a′**) [high magnification of entangled area in lower panel showing spine density from WT (**b**) and cKO mice (**b**′)]. **c** Sholl analysis of apical dendrites shows an increased number of intersections in cKO mice compared to WT mice, indicating an enhanced dendritic arborization in cKO in a region 60–120 µm away from soma and **d** increased cumulative length of dendrites in cKO mice compared to WT mice (WT: *N* = 5 mice, *n* = 10 cells; cKO: *N* = 6 mice, *n* = 11 cells). **e** The density of spines measured in the region of increased dendritic arborization in cKO is reduced when compared WT littermates (*N* = 4 mice each; WT: *n* = 7 cells, cKO: *n* = 8 cells). Analysis of pyramidal cells in the CA1 region of WT (**f**) and cKO (**f**′) mice reveals no change in **g**, arborization of basal and apical dendrites or **h**, total dendritic branch length. Scale bar in **a, f** is 15 µm and in **b** is 5 µm. All values are mean ± SEM; *p ≤ 0.05, **p ≤ 0.01, two-way repeated measures ANOVA (**c, g, h**) and Student’s *t* test (**d, e**)

Neurons in *Bsn*^*ΔEx4*/*5*^ mice were reported to display morphological changes (Sgobio et al. 2010). Based on the observed behavioral phenotype and the physiological alterations at MPP-DG synapses, we next analyzed the morphology of DG granule cells using the Golgi impregnation method (Fig. 7a, a′). Sholl analysis revealed an increased arborization of DG granule cell dendrites in *Bsn* cKO mice [Fig. 7c, *F*(1,9) = 10.43, *p* = 0.0103, two-way repeated measures ANOVA]. Specifically, dendritic complexity was higher in the region 60–120 µm distant from the cell soma [*t*(9) = 3.481, *p* = 0.0069, Student’s *t* test]. The total dendritic branch length was also significantly increased in *Bsn* cKO mice [Fig. 7d, *t*(9) = 2.546, *p* = 0.0314]. Based on these findings, we further investigated the density of spines in the dendritic region between 60–120 µm away from the soma, which was decreased in *Bsn* cKO mice (Fig. 7b′) compared to their WT littermates [Fig. 7b, e; *t*(6) = 3.904, *p* = 0.0079]. To control for the regional specificity of these effects, we also analyzed CA1 pyramidal neurons from WT and *Bsn* cKO mice (Fig. 7f, f′). However, here, no significant genotype effect in number of intersections [Fig. 7g, *F*(1,10) = 0.03, *p* = 0.8671] and total dendritic branch length [Fig. 7h, *F*(1,10) = 0.01, *p* = 0.9193] could be observed.

Finally, as *Bsn*^*ΔEx4*/*5*^ mice display a significantly increased forebrain volume (Angenstein et al. 2007), we compared the brain volume between *Bsn* cKO and WT mice using Manganese-enhanced magnetic resonance imaging (ME-MRI). Our analysis revealed that *Bsn* cKO mice display an increased total brain volume when compared to WT littermates [*t*(8) = 2.761, *p* = 0.0246, Student’s *t* test]. This change mainly attributes to cortical regions [*t*(8) = 3.260, *p* = 0.0115]. In the hippocampus, a tendency towards increased volume is also apparent but does not reach significance level [*t*(8) = 2.228, *p* = 0.0565]. The volume of the cerebellum by contrast [*t*(8) = 0.9236, *p* = 0.3827] remained unchanged by the mutation.

### Lack of maturation-induced decrease in the excitability at the MPP-DG synapse of *Bsn* cKO mice

**Fig. 8:**
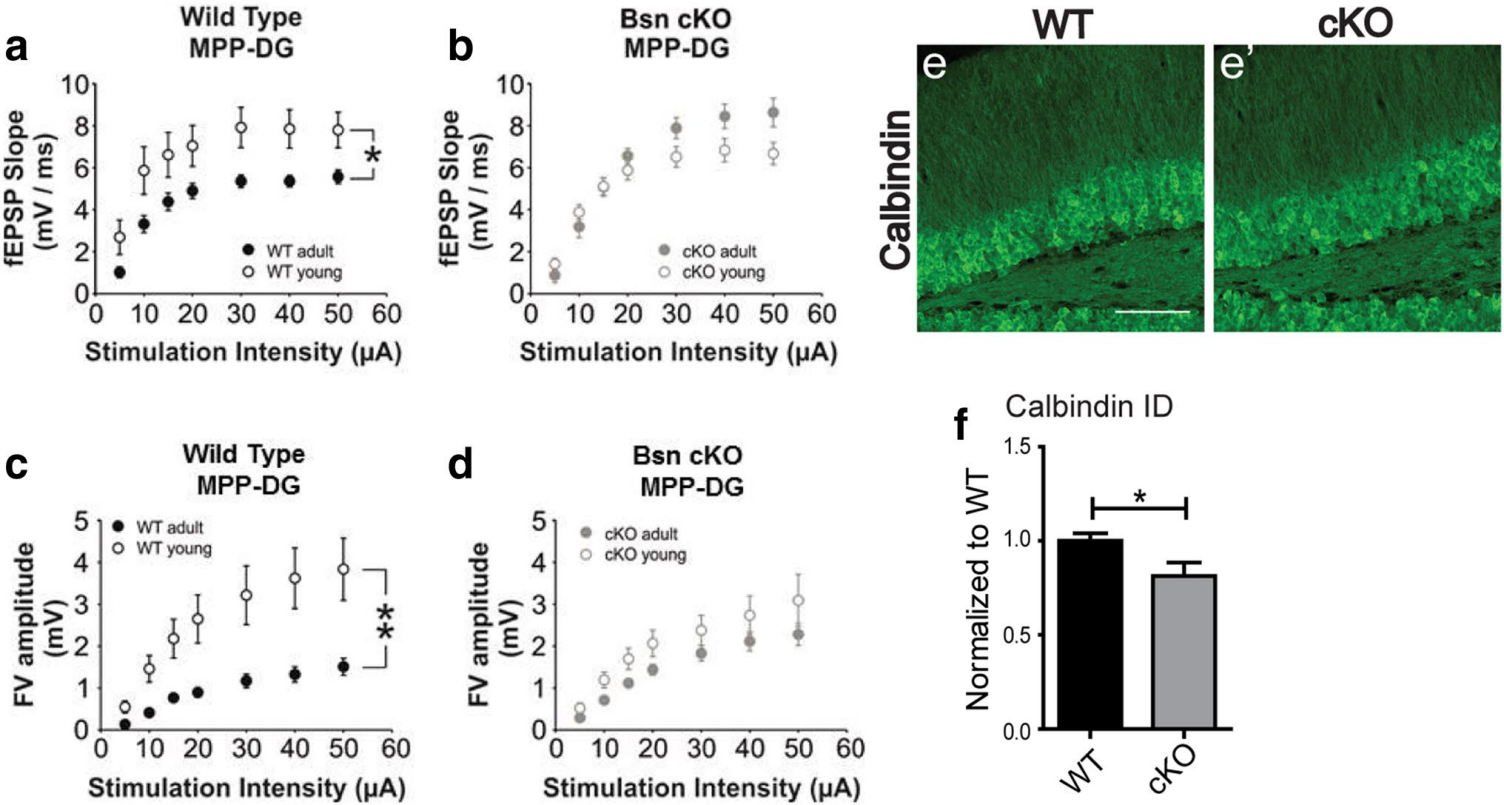
Lack of age-dependent electrophysiological maturation and reduced calbindin levels in the DG of *Bsn* cKO mice. **a** Summary graph showing the age-dependent decrease in baseline excitability in the MPP-DG synapse of WT mice (WT adult: *N* = 4 mice, *n* = 11 slices, WT young: *N* = 5 mice, *n* = 11 slices). **b** By contrast an age-dependent decrease in baseline excitability in the MPP-DG synapse cannot be seen in cKO mice (cKO adult: *N* = 4 mice, *n* = 11 slices, cKO young: *N* = 5 mice, *n* = 11 slices). **c** Summary graph showing a maturation-induced decrease in FV amplitudes in the MPP-DG synapse of WT mice (WT adult: *N* = 5 mice, *n* = 11 slices, WT young: *N* = 5 mice, *n* = 9 slices). **d** By contrast a similar age-dependent decrease in FV amplitudes is lacking at the MPP-DG synapse of cKO mice (cKO adult: *N* = 4 mice, *n* = 9 slices, cKO young: *N* = 5 mice, *n* = 10 slices). Representative dorsal hippocampus sections from WT (**e**) (*N* = 6) and cKO (**e**′) (*N* = 6) mice stained for calbindin as a marker of mature granule cells. **f** Quantification of calbindin labeling (integrated density values) confirms a reduced expression in cKO mice. Scale bar in **e** is 100 µm. All values are expressed as mean ± SEM. *p ≤ 0.05, **p ≤ 0.01, two-way repeated ANOVA followed by post hoc comparison using Fisher LSD Method (**a**–**c**) Student’s *t* test (**f**)

Based on the above findings and previous observations, we hypothesized that lack of Bassoon may impair maturation of the dentate gyrus and, therefore, compared electrophysiological properties of young (~ 4 to 5 weeks) and adult (~ 12 to 16 weeks) WT and *Bsn* cKO mice. At the MPP-DG synapse of WT mice, excitability was reduced during maturation as fEPSP slopes were significantly smaller in adult WT mice than in young WT mice [Fig. 8a, *F*(1, 22) = 7.131, *p* = 0.014, two-way repeated measures ANOVA]. By contrast, high MPP-DG synapse excitability of *Bsn* cKO mice was maintained during maturation, and no significant alteration between I–O curves of the young *Bsn* cKO mice and adult *Bsn* cKO mice became evident [Fig. 8b, *F*(1, 20) = 1.592, *p* = 0.222]. For the SC-CA1 synapse measured in the same slices, there was no significant difference between the I–O curves of young and adult mice of either genotype [Online Resource 6a, b; WT: *F*(1, 23) = 3.758, *p* = 0.065; *Bsn* cKO: *F*(1, 22) = 0.171, *p* = 0.683]. Next, we wondered whether the lack of maturation-induced decrease in fEPSP slopes at the MPP-DG synapse of adult *Bsn* cKO mice was associated with any adaptive presynaptic change. Thus, we compared the FV amplitudes obtained from MPP-DG synapse of young vs. adult WT and *Bsn* cKO mice. Indeed, in WT animals, we observed a significant decrease in FV amplitudes during maturation [Fig. 8c, *F*(1, 18) = 10.275, *p* = 0.005, two-way repeated measures ANOVA]. Similar to unaltered postsynaptic excitability in *Bsn* cKO mice, we observed no significant decrease in presynaptic FV amplitudes during development [Fig. 8d, *F*(1, 18) = 2.479, *p* = 0.133, two-way repeated measures ANOVA]. Finally, analysis of fEPSP slope to FV amplitude ratios showed no significant genotype difference at MPP-DG synapse of young mice confirming that the phenotype is triggered during maturation (WT: 3166.7 ± 204.9%, *Bsn* cKO: 3014.6 ± 128.8%; *U* = 1848.0, *p* = 0.616, Mann–Whitney *U* test). Based on these electrophysiological findings, we further tested for the expression of different maturation markers in the DG. First, we analyzed the expression of calbindin as a marker for mature granular cells (Fig. 8e, e′). In the granule cell layer (GCL) of *Bsn* cKO mice, immunohistochemical labeling for calbindin (integrated density values) was reduced, when compared to WT mice [Fig. 8f, *t*(10) = 2.274, *p* = 0.0462, Student’s *t* test], resembling the effect in constitutive *Bsn*^*ΔEx4*/*5*^ mutants (Dieni et al. 2015).

### Increased expression of markers for immature granule cells and neurogenesis in the DG of *Bsn* cKO mice

**Fig. 9:**
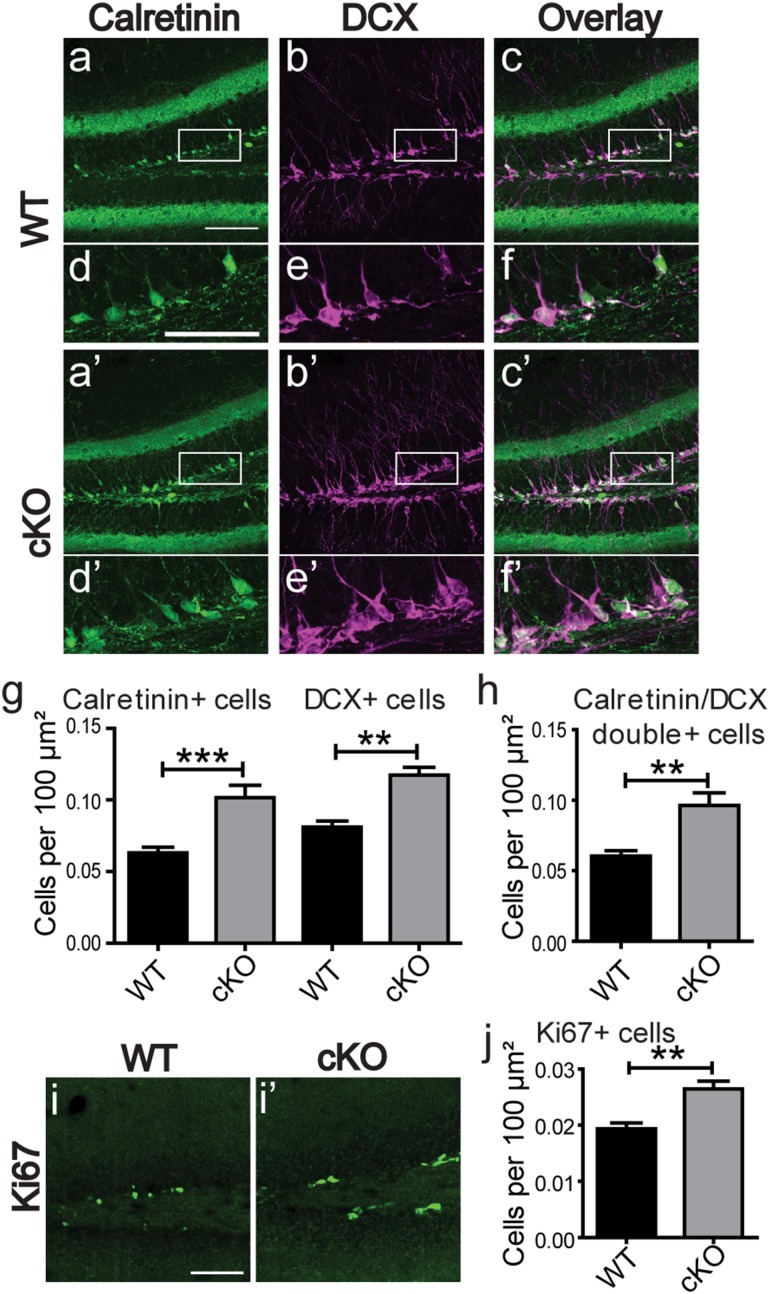
Enhanced neurogenesis and increased numbers of immature granule cells in the DG of *Bsn* cKO mice. Representative dorsal hippocampus sections from WT (**a, b**) (*N* = 5) and cKO (**a**′, **b**′) (*N* = 6) mice were stained for calretinin and doublecortin (DCX) as markers of immature granule cells. Higher magnification of entangled area in lower panel depicting an increased labeling of both markers in cKO mice (**d**′, **e**′), when compared to WT mice (**d, e**). Overlay images showing the calretinin/DCX double positive cells in WT (**c, f**) and cKO mice (**c**′, **f**′). **g**, The densities of immature cells positive for calretinin or DCX, as well as **h**, double positive cells are significantly increased in cKO mice. Representative dorsal hippocampus sections from WT (**i**) (*N* = 5) and cKO (**i**′) (*N* = 5) mice stained for proliferative marker Ki67 in DG and **j**, increased number of Ki67 positive cells in cKO mice, indicating an increased neurogenesis. Scale bar in **a, i** is 100 µm and **d** is 50 µm. All values are expressed as mean ± SEM. **p ≤ 0.01, ***p ≤ 0.001, two-way repeated ANOVA followed by post hoc comparison using Bonferroni posttest (**g**) and Student’s *t* test (**h, j**)

Next, we assessed whether the deficit in maturation is also reflected by the presence of increased numbers of newly generated cells. To this end, we stained for cells in an early stage of differentiation using calretinin and doublecortin (DCX) as markers. We found that the number of cells positive for either calretinin (Fig. 9a, d, a′, d′; *p* < 0.001, Bonferroni posttest) or DCX (Fig. 9b, e, b′, e′; *p* < 0.01) were strongly increased in the granule cell layer of *Bsn* cKO mice [Fig. 9g, *F*(1,9) = 18.86, *p* = 0.0019, two-way repeated measures ANOVA]. The number of cells double positive for calretinin and DCX (Fig. 9c, f, c′, f′) were also significantly increased (Fig. 9h, *t*(9) = 3.501, *p* = 0.0067), but the proportion of calretinin-positive cells that were also positive for DCX were not changed between the genotypes (WT: 74.33 ± 2.662%; *Bsn* cKO: 81.06 ± 3.76%). We finally investigated the rate of neurogenesis, as enhanced neurogenesis had been described previously for *Bsn*^*ΔEx4*/*5*^ mutants (Heyden et al. 2011) and this might contribute to the increased number of immature cells in DG of cKO mice. To this end, we used the Ki67 antigen as a marker to identify proliferating cells in DG (Fig. 9i, i′). We observed a significant increment of Ki67-positive cells in GCL of *Bsn* cKO mice as compared to WT mice [Fig. 9j and Online Resource 7; *t*(8) = 4.102, *p* = 0.0034] indicating indeed an enhanced neurogenesis in *Bsn* cKO mice.

## Discussion

To genetically dissect Bassoon functions at different types of synapses and to investigate its role in behavioral and network functions, we generated *Bsn* cKO mice lacking Bassoon at excitatory forebrain synapses. These mice show an increase in contextual fear memory and increased preference for the novel object location in a spatial discrimination/pattern separation task. These behavioral changes are associated with enhanced neurogenesis, an increased expression of markers for immature granule cells in the DG and a concomitant preservation of juvenile neuronal excitability at MPP-DG synapses. As one of the central organizers of the presynaptic CAZ, Bassoon thus seems to control synaptic properties related to specific forms of memory formation in the adult central nervous system, and to be involved in the structural and functional maturation of the DG.

Constitutive *Bsn* mutant mice have been generated previously, but the analysis of genuine Bassoon functions in control of behavior has been hampered by occurrence of epileptic seizures and sensory deficits in these animals (Altrock et al. 2003; Dick et al. 2003; Khimich et al. 2005). This caveat has been overcome with the generation of *Bsn* cKO mice, which show a loss of Bassoon expression selectively from the VGLUT1-positive terminals of excitatory forebrain neurons. By contrast, the activation of the conditional allele with an *Emx1*-Cre driver mouse spared VGAT-positive terminals of inhibitory interneurons and putative neuromodulatory afferences (Gasbarri et al. 1997; Picciotto et al. 2012) bearing residual Bassoon labeling in VGAT- and VGLUT-negative terminals in the molecular layer of the DG (Fig. 2). Although we cannot exclude that susceptibility for seizure generation might be elevated under certain conditions (chemically induced epilepsy models, etc.), *Bsn* cKO mice did not show any overt spontaneous seizures, survived well and without any difference from their WT littermates (Online Resource 1e). Furthermore, they performed equally or even superior to their WT littermates in several visual and auditory tasks (Fig. 3, 4 and Online Resource 2,4).

The behavioral analysis of *Bsn* cKO mice showed an enhanced contextual fear memory without alterations in auditory cued fear conditioning suggesting a change of hippocampus-dependent, but not amygdala-dependent fear memory processing (Maren et al. 2013). We could not observe any change in shock sensitivity, exploratory or anxiety-related behavior or in response learning in an active avoidance task. On the other hand, preliminary data on improved performance during initial training sessions of a frequency-modulated tone discrimination task (Tischmeyer, Annamneedi et al. unpublished) point to a possible change in auditory cortex-dependent learning of *Bsn* cKO mice. In contrast, when subjecting *Bsn* cKO mice to the Morris water maze task, we found no evidence for a change in spatial learning or re-learning. We further employed a novel object location task to assess intrinsically motivated one-trial spatial learning. Since both WT and *Bsn* cKO performed well in the Morris water maze task we decided to render the novel object location task rather difficult by changing object location by 90 degrees from the original position and testing memory after 24 h. In fact, WT showed relatively poor performance, whereas *Bsn* cKO mice showed a tendency to prefer the novel object location in this paradigm. More importantly, *Bsn* cKO mice displayed a significantly increased preference than WT mice for the novel location in a related spatial discrimination/pattern separation task. Metric processing of object location can be utilized to examine pattern separation in mice (Bekinschtein et al. 2013; van Hagen et al. 2015). Again, we employed a rather difficult paradigm with a small distance between the targeted objects and WT mice indeed displayed avoidance rather than approach as has previously been observed when animals failed to learn such tasks (Bekinschtein et al. 2013). *Bsn* cKO mice on the other hand showed a strong selective approach to the novel location in this learning paradigm. We cannot exclude that the different responding (approach in *Bsn* cKO, avoidance in WT) in this task may involve functions other than the ability for spatial discrimination and pattern separation. However, we found no evidence for altered novelty responding or anxiety-related behavior in the *Bsn* cKO mice. The converging evidence from contextual fear conditioning and spatial discrimination/pattern separation tasks thus led us to further investigate potential changes in hippocampal physiology in *Bsn* cKO mice.

In fact, in hippocampal slice preparations we observed higher baseline excitability and increased fEPSP-to-FV amplitude ratio at MPP-DG synapses that may explain the behavioral observations. These physiological changes are likely to increase granule cell responsiveness to stimulation during the acquisition and/or retrieval of contextual and spatial tasks (Saxe et al. 2006; Deng et al. 2009) and are also in good agreement with a role of the DG in contextual fear memory processing (Lee and Kesner 2004; Kheirbek et al. 2013; Liu et al. 2012). They are also in agreement with the critical role of the DG in spatial discrimination (Hunsaker et al. 2008; Clelland et al. 2009) and pattern separation (Gilbert et al. 1998; Leutgeb et al. 2007) as well as the responsiveness of this function to stimulation of DG function (Bekinschtein et al. 2013).

The observed increases of FV and EPSP at the SC-CA1 synapse, on the other hand, were not accompanied by any change in fEPSP-to-FV ratios. This is indicative of a normalized baseline transmission at this synapse, which might be due to postsynaptic adaptive mechanisms rescuing this effect in SC-CA1 pathway. Nevertheless, increased excitability of the Schaffer collateral pathway may also affect hippocampal information processing during memory tasks.

Strikingly, we did not find any change in LTP in DG or CA1 under any of the applied stimulation protocols, indicating that expression of Bassoon per se is not required for the induction of this form of neural plasticity. These data contrast previous findings of altered LTP at CA1 synapses in *Bsn*^ΔEx4/5^ mice (Sgobio et al. 2010). Considering the role of Bassoon in regulated neurotransmitter release (Altrock et al. 2003; Gundelfinger et al. 2016) and the importance of the GABAergic system in epilepsy (Treiman 2001), we suggest that absence of Bassoon from inhibitory synapses during network development and/or epileptic seizure activity may contribute to impaired long-term plasticity in constitutive *Bsn* mutants. Epilepsy-induced changes, sensory impairments or disturbance of GABAergic interneuron function may also have been responsible for deficits of *Bsn*^*ΔEx4*/*5*^ mice in active avoidance learning (Ghiglieri et al. 2010) that we could not recapitulate with *Bsn* cKO mice in the current study. On the other hand, we cannot rule out that blockade of GABAergic transmission by addition of picrotoxin, which is a prerequisite for reliable LTP induction in the MPP-DG (Arima-Yoshida et al. 2011), could have masked a potential genotype difference (Sahay et al. 2011). Clearly, it will be interesting to address the specific role of Bassoon in GABAergic interneuron functions in the future.

High excitability as well as expanded dendrites with reduced spine density are the typical properties of young immature DG granule cells (Ge et al. 2007; Schmidt-Hieber et al. 2004; Spampanato et al. 2012). Similarly, we observed an about twofold increase in dendrite branching and accompanying decrease of spine density (~ twofold reduction) in the DG of *Bsn* cKO mice. These might reflect a compensatory response to the increased cellular excitability. However, as these changes occur in a dendritic region more than 60 µm away from the soma it can be expected that the relative weight of inputs to the DG, i.e., perforant path vs. commissural path, may be altered. The processing of information regarding object context or location involves the MPP (Eichenbaum et al. 2007) and DG granule cells are critical components of contextual memory engrams (Liu et al. 2012; Ramirez et al. 2013) with the size of activated DG granule cells ensembles correlating with context memory strength (Stefanelli et al. 2016). Thus, an enhanced excitability and associated structural changes in the medial molecular layer of DG might well account for the altered performance of *Bsn* cKO mice in spatial discrimination/pattern separation and contextual fear conditioning tasks. Importantly, comparable morphological changes were not observed in the CA1, indicating that altered neuronal development in the DG of cKO mice might be of particular relevance for their physiological and behavioral phenotype.

In fact, immature granule cells in the rodent DG are involved in the formation of contextual fear memories and in performance in pattern separation tasks (Saxe et al. 2006; Clelland et al. 2009) and Nakashiba et al. (2012) have suggested that different populations of granule cells in the DG, i.e., young immature and older granule cells mediate different aspects of pattern analysis tasks. Accordingly, mice with elevated adult hippocampal neurogenesis are improved in differentiating the overlapping context representations (Sahay et al. 2011).

Our physiological data suggest that the increased excitability of the adult DG may reflect similar changes in the perforant pathway-to-dentate gyrus circuit in *Bsn* cKO mice. In fact, we also observed an altered expression of maturation markers calbindin, calretinin and doublecortin in DG granule cell layer of these animals (Hagihara et al. 2013; Ming and Song 2011; Spampanato et al. 2012). Reduced calbindin staining intensity in granule cells has been associated with increased excitability of DG (Magloczky et al. 1997). The reduced calbindin staining, which was seen in both *Bsn* cKO mice (this study) and in *Bsn*^*ΔEx4*/*5*^ mice (Dieni et al. 2015), can occur as a consequence of *Bsn* gene ablation independently of epileptiform activity. We also demonstrate a profound increase in the density of calretinin and doublecortin labeled cells in the granule cell layer of *Bsn* cKO mice, which may indicate an increased number of DG granule cells in an early postmitotic differentiation stage (von Bohlen und Halbach 2007). Finally, we show an increase of Ki67 positive cells in *Bsn* cKO mice, suggestive of an increase in neurogenesis, which has previously also been observed in *Bsn*^*ΔEx4*/*5*^ mice (Heyden et al. 2011).

An induction of neurogenesis through epileptiform activity in *Bsn* cKO mice is highly unlikely, since in contrast to *Bsn*^*ΔEx4*/*5*^ mice, they did not display any overt seizure activity or increased lethality. Then, how can the lack of Bassoon, a protein that is expressed in post migratory neurons, enhance neurogenesis and reduce maturation markers in the DG? In the wild-type brain, Bassoon transcripts occur in late embryonic development and highest levels are seen at postnatal day 21, particularly in the pre-granule cell layer and differentiating granule cells of the DG (Zhai et al. 2000). Given the expression onset of *Emx1* at embryonic day 10.5 (Gorski et al. 2002), we can expect that Bassoon is not expressed in excitatory synapses of cKO mice at any stage of development. This might lead to a deficit in axonal maturation and hyperexcitability, as indicated by the increase in FV in the MPP. Noteworthy, stimulation of the entorhinal cortex has previously been shown to stimulate hippocampal neurogenesis (Stone et al. 2011).

Bassoon supports the assembly of presynaptic boutons from preassembled Piccolo-Bassoon transport vesicles (PTVs) (Zhai et al. 2001; Shapira et al. 2003; Dresbach et al. 2006). By interacting with dynein light chain DLC-1, it serves as one cargo adaptor to microtubules and disturbance of these interactions attenuates transport of PTVs in young axons (Fejtova et al. 2009). Thus, Bassoon knockout may lead to a shortage of material for synapse assembly and maturation. In line with this, an interaction of Bassoon with the autophagosome factor Atg5 and requirement for synaptic autophagy has been reported recently (Okerlund et al. 2017). Autophagy is critical for presynaptic development and homeostasis (Vijayan and Verstreken 2017) as well as axonal pruning during development (Song et al. 2008), and the lack of decrease in FV amplitude and DG excitability during the postnatal development of *Bsn* cKO mice may well result from a disturbance of these functions. Another, not mutually exclusive scenario considers that the developmental gene expression program is affected in Bassoon-deficient neurons. Bassoon can bind the chromatin-modifying transcriptional co-repressor CtBP1, a well-known neurodevelopmental regulator (Chinnadurai 2007), and thereby control synapto-nuclear shuttling of the repressor (Ivanova et al. 2015). Lack of Bassoon can shift the equilibrium towards a higher CtBP1 concentration in the nucleus and may thus repress transcriptional programs required for controlling axonal excitability. In fact, the increased FV was not restricted to the MPP, but also observed in the SC pathway. However, while SC-CA1 transmission was balanced by synaptic adaptation, an increase in baseline transmission was only observed in the DG, maybe due to the existence of a neurogenic niche and activity-induced enhancement of adult neurogenesis.

Together, our data document the importance of Bassoon in the postnatal development of the perforant path-to-DG circuit and in contextual and spatial learning in the adult. The altered performance of *Bsn* cKO mice in contextual fear conditioning and a spatial discrimination/pattern separation task appears to be related to an increased excitability of the DG granule cells, but apparently not to any change in synaptic plasticity. It is important to note that this finding does not merely reflect an overall delay of development as adolescent mice have been reported with normal or even reduced context fear memory (Pattwell et al. 2011; Akers et al. 2012). We observed morphological and physiological alterations as well as changes of expression of differentiation markers in the DG of adult *Bsn* cKO mice that resemble characteristics of immature granule cells. Disturbed maturation of the DG has been identified as a critical process in schizophrenia and depression (Hagihara et al. 2013) and future studies will have to address the potential involvement of Bassoon-mediated cellular processes in these psychopathologies.

## Electronic supplementary material

Below is the link to the electronic supplementary material.

Supplementary material 1 (PDF 1167 KB)
