# Supplementary material for: Ablation of the presynaptic organizer Bassoon in excitatory neurons retards dentate gyrus maturation and enhances learning performance
Source: Brain Struct Funct. 2018 Jun 18;223(7):3423–45. doi: 10.1007/s00429-018-1692-3 (PMC6132633; doi:10.1007/s00429-018-1692-3)
Supplement: Supplementary file 1 — Supplementary material 1 (PDF 1167 KB) [file 429_2018_1692_MOESM1_ESM.pdf]

Ablation of the presynaptic organizer Bassoon in excitatory neurons retards dentate gyrus maturation and enhances learning performance

Anil Annamneedi[1,#], Gürsel Caliskan[2,#], Sabrina Müller[1], Dirk Montag[3], Eike Budinger[4,7], Frank Angenstein[5,11], Anna Fejtova[1,6,7,8], Wolfgang Tischmeyer[7,9], Eckart D. Gundelfinger[1,7,10,11] and Oliver Stork[2,7*]

[1]Department of Neurochemistry and Molecular Biology, Leibniz Institute for Neurobiology, Magdeburg, Germany.

[2]Department of Genetics and Molecular Neurobiology, Institute of Biology, Otto-von-Guericke-University, Magdeburg, Germany.

[3]Neurogenetics Laboratory, Leibniz Institute for Neurobiology, Magdeburg, Germany.

[4]Department Systems Physiology of Learning, Leibniz Institute for Neurobiology Magdeburg, Magdeburg, Germany.

[5]Special Laboratory Noninvasive Brain Imaging, Leibniz Institute for Neurobiology, Magdeburg, Germany.

[6]RG Presynaptic Plasticity, Leibniz Institute for Neurobiology, Magdeburg, Germany.

[7]Center for Behavioral Brain Sciences (CBBS), Magdeburg, Germany.

[8]Department of Psychiatry and Psychotherapy, University Hospital, Friedrich-Alexander-University Erlangen-Nuremberg, Erlangen, Germany.

[9]Special Laboratory Molecular Biological Techniques, Leibniz Institute for Neurobiology, Magdeburg, Germany

[10]Molecular Neuroscience, Medical School, Otto von Guericke University, Magdeburg, Germany.

[11]Functional Neuroimaging Group, German Center for Neurodegenerative Diseases, Magdeburg, Germany.

# Authors contributed equally

Anil Annamneedi: Anil.Annamneedi@lin-magdeburg.de

Gürsel Caliskan: guersel.caliskan@ovgu.de

Sabrina Müller: Sabrina@mueller-rodgau.net

Dirk Montag: Dirk.Montag@lin-magdeburg.de

Eike Budinger: Eike.Budinger@lin-magdeburg.de

Frank Angenstein: Frank.Angenstein@dzne.de

Anna Fejtova: Anna.Fejtova@uk-erlangen.de

Wolfgang Tischmeyer: Wolfgang.Tischmeyer@lin-magdeburg.de

Eckart D. Gundelfinger: Eckart.Gundelfinger@lin-magdeburg.de

for correspondence

Oliver Stork: oliver.stork@ovgu.de, Telephone: +49(391)-67 55100, Fax: +49(391)-67 55102

Supplementary Information

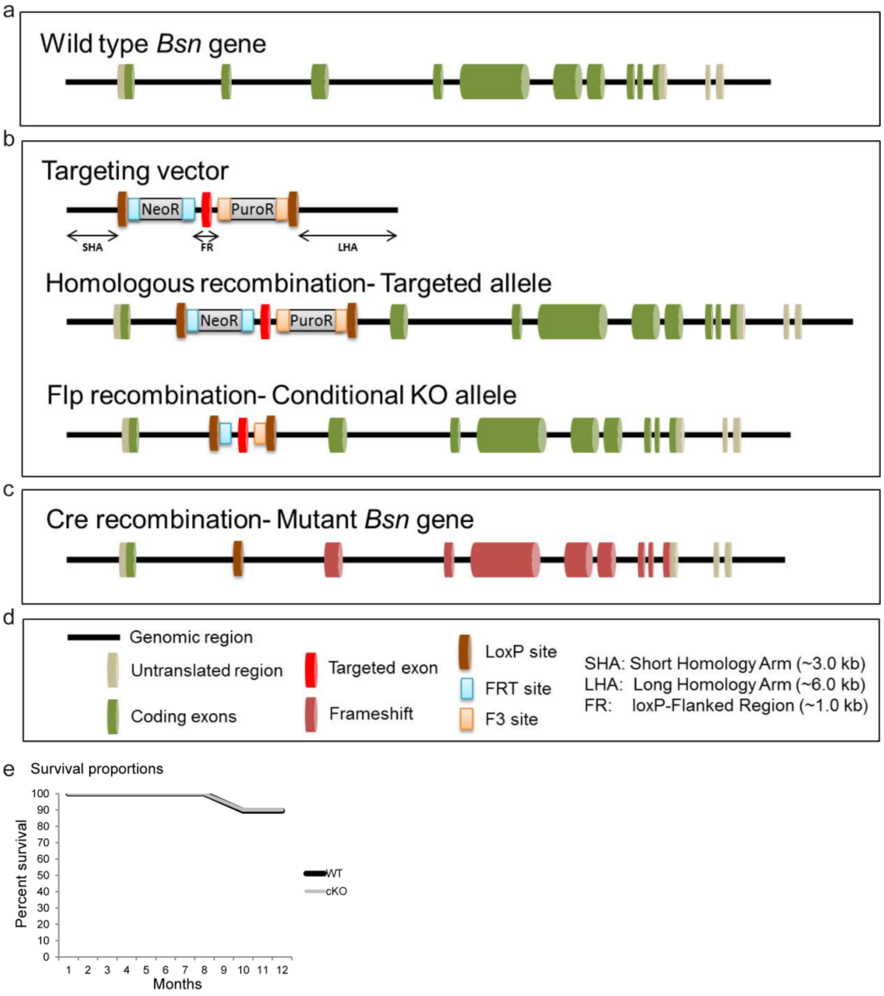

Online Resource 1. Generation of *Bsn* cKO mice. a, Schematic representation of the wild type *Bsn* gene containing 12 exons. **b,** Gene targeting strategy for generating *Bsn2*$^{lx/lx}$ mice (adopted from Taconic Artemis GmbH). The targeting vector contains FRT flanked to Neomycin resistance and F3 site flanked to Puromycin resistance selection markers, along with loxP sites and this was inserted into the targeting allele, flanking the exon 2, by homologous recombination. Flp-mediated recombination results in a conditional knockout (KO) allele. *Bsn2*$^{lx/lx}$ mice (containing the conditional KO allele) are then crossed with Cre driver mice, (B6.129S2-Emx1$^{tm1(cre)Krj}$, The Jackson Laboratory, Gorski et al., 2002) allowing Cre recombination, which results in a deletion of exon 2 of the *Bsn* gene. This causes a frameshift and a premature stop in exon 3 **(c)**. Mice expressing Cre recombinase are termed *Bsn* cKO mice (*Bsn2*$^{lx/lx}$*Emx1*$^{Cre/+}$) and littermates without Cre recombinase are referred to as WT mice (*Bsn2*$^{lx/lx}$*Emx1*$^{+/+}$). **d,** Explanations for the abbreviations and symbols used in the schematic representation. **e,** Survival plot indicating comparable survival rate of cKO (N=20) mice compared to WT (N=19) mice.

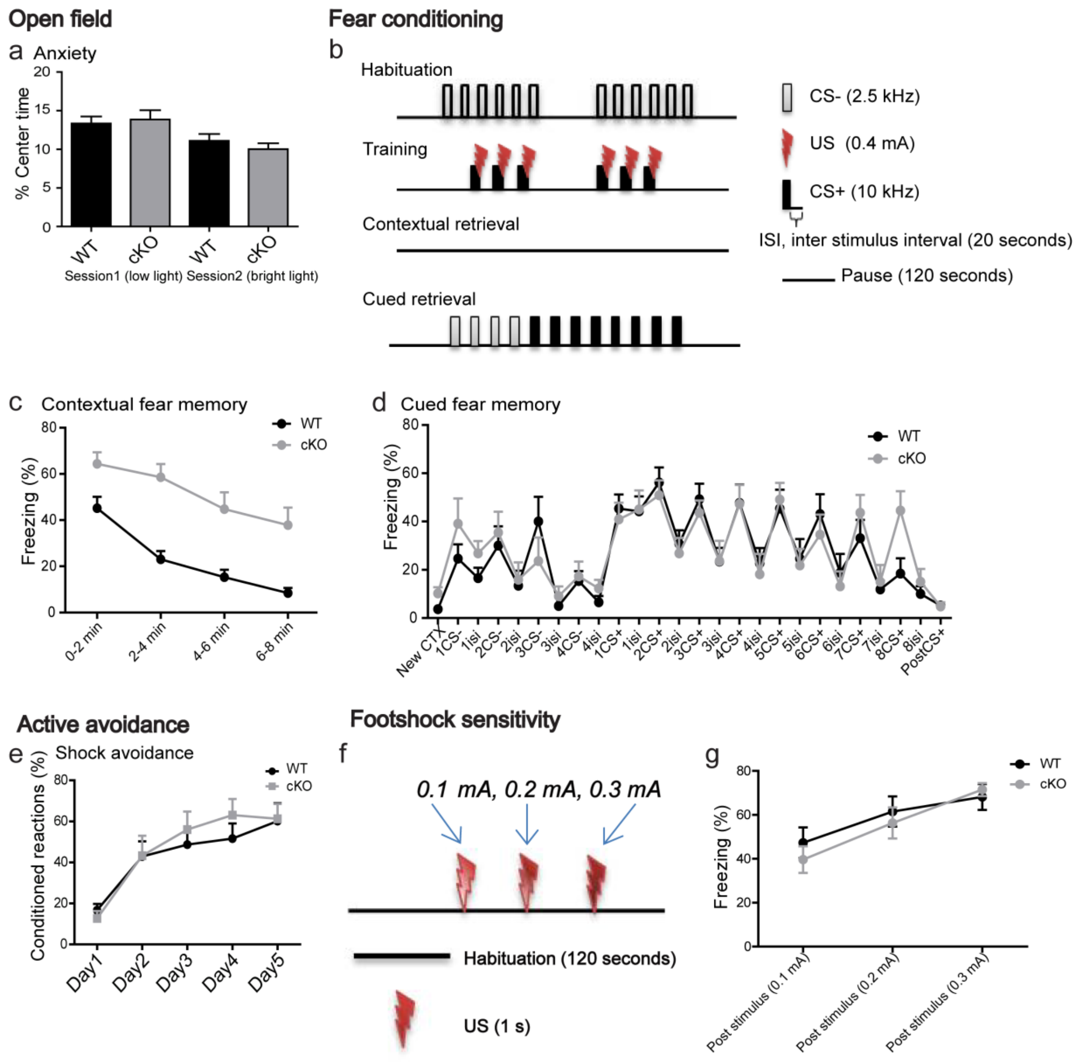

Online Resource 2. Behavior of *Bsn* cKO mice. a, WT and cKO mice spend similar time in the center of an open field arena in low light and bright light conditions, indicating comparable anxiety levels (WT: N=24; cKO: N=19). **b,** Schematic representation of the fear conditioning paradigm. Mice (WT: N=13 mice; cKO: N=11 mice) are first habituated to training context and to neutral tones (CS-) (2 sets of six presentations of CS- stimuli, each separated with 120 sec of pause). During training, mice encounter 2x 3 presentations of conditional stimuli (CS+), each co-terminating with an electric foot shock. Memory towards the shock context is tested 24 hrs after training in a single session without tone exposure. Auditory-cued memory is tested in neutral context with an exposure of sets of 4 CS- followed by 8 CS+. **c,** Analysis of contextual freezing during the retrieval session, analyzed in four 2 min bins. Throughout the session freezing in *Bsn* cKO mice is increased compared to WT littermates (genotype effect: F(1,22)=20.81, p=0.0002; genotype x session duration interaction: F(3,66)=2.82, p=0.0454). **d,** WT and cKO mice show comparable freezing in the neutral context and during presentation of each CS- and CS+ (genotype effect: F(1,22)=0.11, p=0.7420). Both groups exhibit profound freezing to CS+ stimulus compared to CS- (stimulus effect: F(25,550)=13.32, p<0.0001). **e,** Mean number of avoidance responses (expressed as %) per day for each group (WT: N=11; cKO: N=9) during the active avoidance test. cKO and WT mice show comparable learning curves over the sessions. **f,** Schematic representation of the foot shock sensitivity test (WT: N=11, cKO: N=9) **g,** A significant effect of shock intensity is observed (F(2,36)=25.83, p<0.0001), but no main effect of genotype (F(1,18)=0.16, p=0.6897,) or shock intensity x genotype interaction (F(2,36)=1.21, p=0.3088). Further behavior scored are flinching (54.5 % of WT and 22.2 % of cKO (n=9) show flinching behavior during the 0.1mA stimulus; p=0.1421, χ^2 test) and vocalization (at 0.1mA st 18.2 % of WT, 11.1 % of cKO, p=0.6595; at 0.2mA stimulus 54.5 % of WT, 33.3 % of cKO, p=0.3428; at 0.3mA stimulus 90.9 % of WT, 77.8 % of cKO, p=0.4132; mice exhibit vocalization), which are not different between genotypes. All values are mean ± SEM; ****P* ≤ 0.001, two-way repeated measures ANOVA (**a,c,d,e,g**) and Chi-square test.

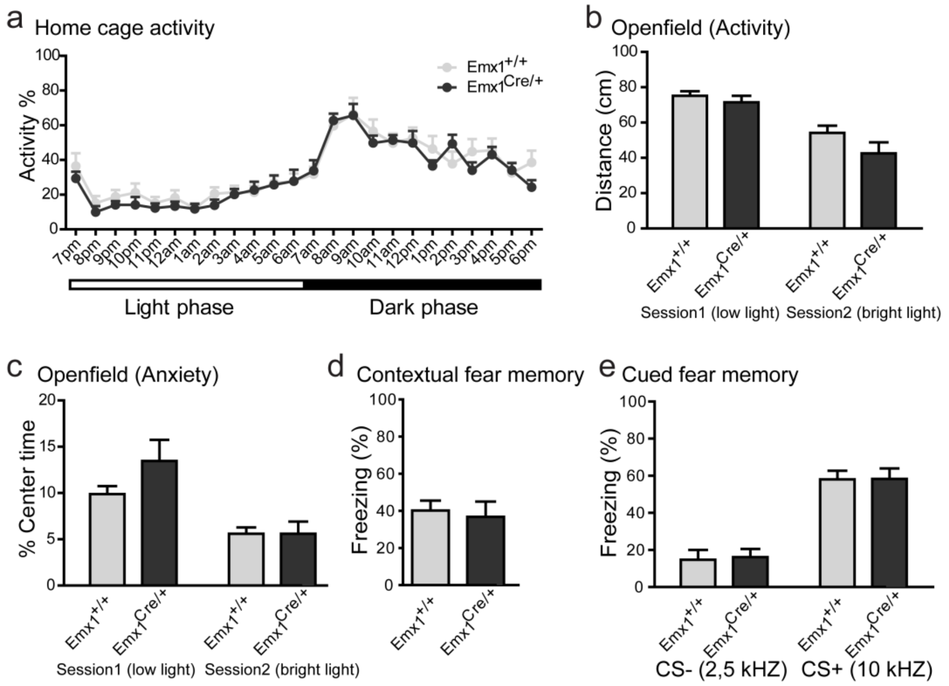

Online Resource 3. No change in the behavior of *Emx1* control groups. a, In home cage activity analysis (*Emx1*$^{+/+}$: N=8, *Emx1*$^{Cre/+}$: N=9), a significant effect of the day time is observed with both groups showing increased activity during the 12 hrs of dark phase of the cycle (F(23,345)=32.55, p<0.0001). No significant genotype effect (F(1,15)=0.37, p=0.5512) and no interaction with day time (F(23,345)=0.92, p=0.5780) is evident. 12 hrs - average activity values are also not changed between the groups, dark phase (*Emx1*$^{+/+}$: 46.85 ± 6.04%; *Emx1*$^{Cre/+}$: 44.54 ± 2.75%, t(15)=0.3621, p=0.7224) and light phase (*Emx1*$^{+/+}$: 21.22 ± 2.89%; *Emx1*$^{Cre/+}$: 17.94 ± 2.58%, t(15)=0.8511, p=0.4081). **b,** Open field analysis (*Emx1*$^{+/+}$: N=9, *Emx1*$^{Cre/+}$: N=9) of distance travelled using low light and bright light test sessions reveals a significant effect of the test session (F(1,16)=51.16, p<0.0001, two-way repeated measures ANOVA), but no genotype effect (F(1,16)=2.28, p=0.1508) or genotype x session interaction (F(1,16)=1.28, p=0.2743). **c,** In addition, is a significant effect of the test session on the center exploration of the open field (F(1,16)=23.70, p=0.0002), but no genotype effect (F(1,16)=1.26, p=0.2783) or genotype x session interaction (F(1,16)=2.07, p=0.1695) can be observed. **d,** Mice (*Emx1*$^{+/+}$: N=9, *Emx1*$^{Cre/+}$: N=9) tested in combined contextual and cued fear conditioning paradigm display comparable pre-training baseline freezing levels and increased freezing levels in the shock context 24 hrs later. This results in a significant session effect (F(1,16)=47.68, p<0.0001), but no genotype effect (F(1,16)=0.07, p=0.7986) or genotype x session interaction (F(1,16)=0.19, p=0.6718). **e,** Both genotypes show low freezing levels to the non-conditioned auditory test stimulus (CS-), but strong freezing to the CS+ (effect of test stimulus: F(1,16)=75.77, p<0.0001). No change is evident for effect of genotype (F(1,16)=0.03, p=0.8728) or genotype x test stimulus interaction (F(1,16)=0.01, p=0.9114). All values are mean ± SEM; two-way repeated measures ANOVA (**a,b,c,e**) and Student's t-test (**d**).

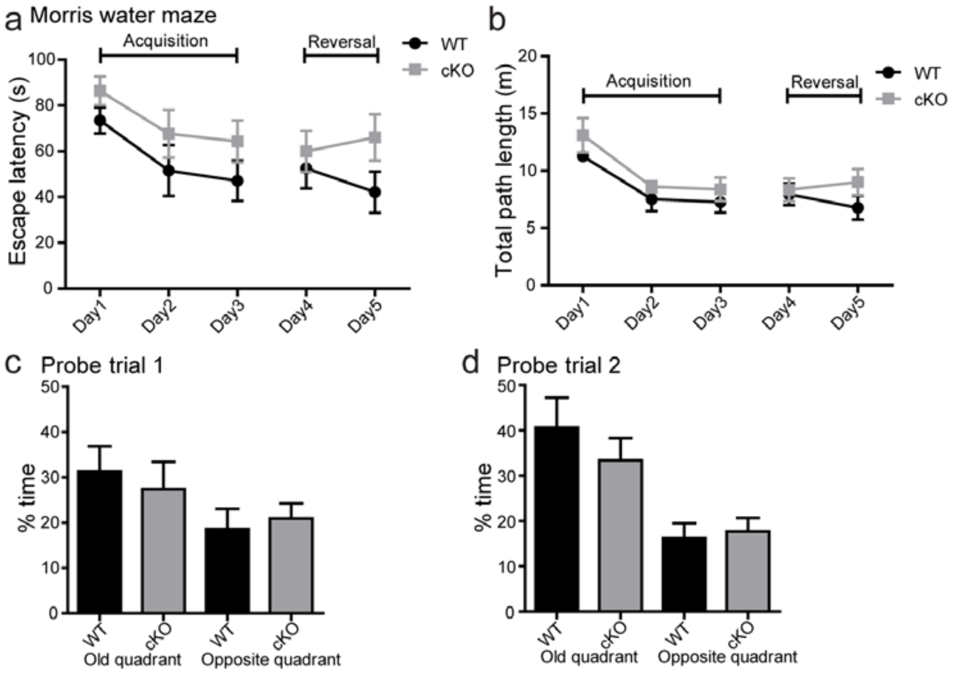

Online Resource 4. *Bsn* cKO mice display normal spatial learning and memory. Normal spatial learning and memory is observed in *Bsn* cKO (N=9), compared to WT (N=9) littermates in hidden platform version of Morris water maze. Both cKO and WT mice display comparable escape latency **(a)** and total path length covered **(b)** to find the hidden platform during 3 days of acquisition phase and 2 days of reversal phase. **c, d,** Both groups spend comparable high time (%) in the old platform quadrant and lower levels in the opposite platform quadrant during the probe trials. All values are mean ± SEM; two-way repeated measures ANOVA (**a,b**) and Student's t-test (**c,d**).

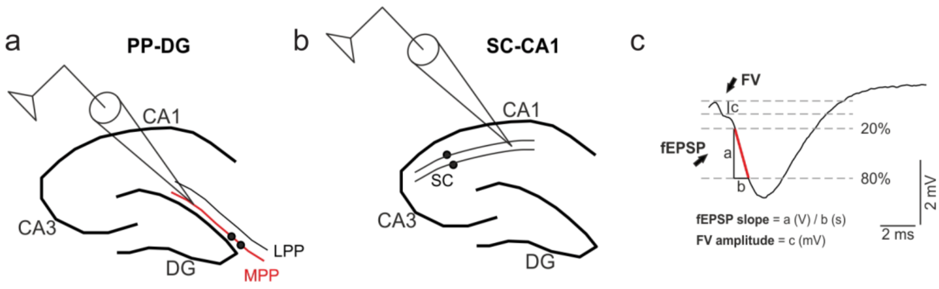

Online Resource 5. Scheme describing experimental conditions and analysis for electrophysiological recordings. Parasagittal brain slices including dorsal hippocampus were prepared for the analysis of electrophysiological parameters in the WT and *Bsn* cKO mice. **a,** For electrophysiological recordings from MPP-DG synapse, the recording electrode was placed in the mid-molecular layer. A bipolar tungsten stimulation electrode (two black dots) was placed at MPP ~300µm distance to recording electrode. **b,** For electrophysiological recordings from Schaffer collateral-CA1 (SC-CA1) synapses, the recording electrode was positioned at the stratum radiatum of CA1 subregion and the stimulation of SC was succeeded by placing a bipolar tungsten electrode at the stratum radiatum of proximal CA1 subregion (two black dots). **c,** A typical response to MPP or SC stimulation showing a presynaptic fiber volley (FV) followed by dendritic excitatory postsynaptic field potential (fEPSP). The slope of the fEPSP was used to determine efficacy of synaptic transmission. The slope (V/s) was calculated using the data points at 20% and 80% of maximum fEPSP amplitude.

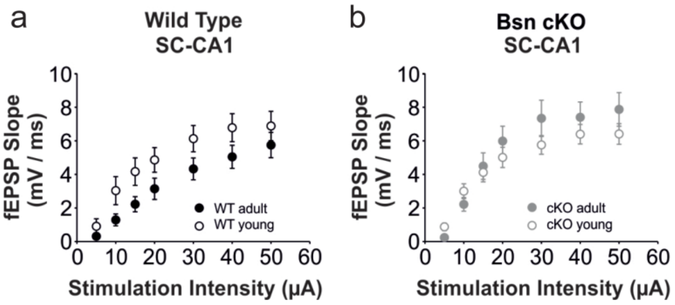

Online Resource 6. Unaltered baseline excitability in the SC-CA1 synapse of young and adult mice. a, Summary graph showing an insignificant trend towards decreased excitability in the SC-CA1 synapse of adult WT mice in comparison to young WT mice (WT adult: N = 4 mice, n = 13 slices, WT young : N = 5 mice, n = 11 slices). **b,** Summary graph showing no alteration in the excitability of SC-CA1 synapse between the adult and young *Bsn* cKO mice (cKO adult: N = 4 mice, n = 13 slices, cKO young : N = 5 mice, n = 12 slices). All values are expressed as mean ± SEM. ns = not significant. (Two-way repeated measures ANOVA followed by posthoc comparison using Fisher LSD Method). SC: Schaffer collaterals, CA1: Cornu ammonis 1.

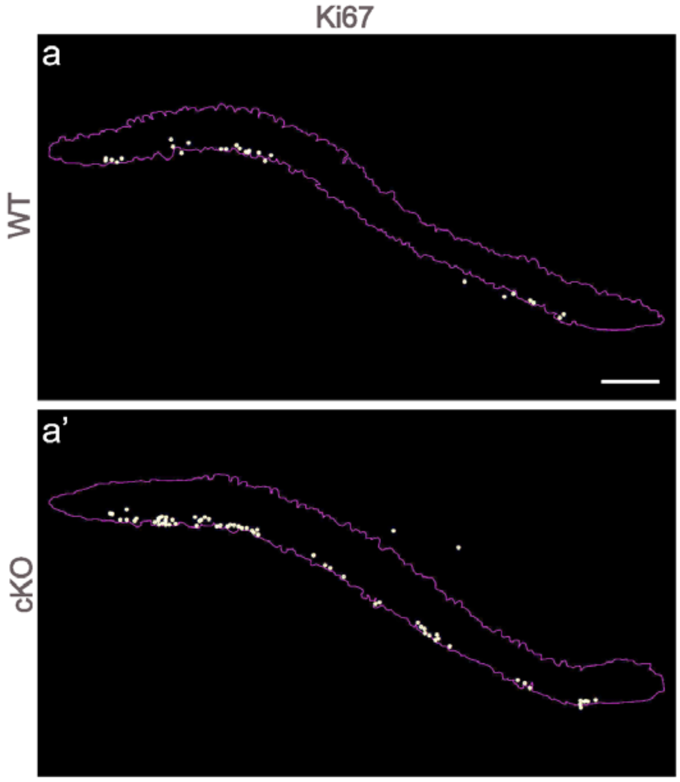

Online Resource 7. Detection of neurogenesis in the DG. Representative example of a reconstructed granule cell layer (suprapyramidal blade) and labeling of cells positive for Ki67 in a *Bsn* WT **(a)** and a *Bsn* cKO mouse **(a')**. The granule cell layer was outlined based on compact DAPI staining. Ki67 positive cells in the subgranular zone were quantified and compared between genotypes. An increased number of Ki67 positive cells is evident in cKO mice compared to WT. Scale bar in **a** is 100 µm.

Online Resource 8. Overview tables of total area analyzed under different biochemical marker experiments and number of cells counted per marker.

Table 1: Absolute values for Calretinin and DCX staining

Genotype	Total number of ROIs considered per genotype	Total number of Calretinin positive cells counted per animal	Total number of DCX positive cells counted per animal
**WT**	39	163.8 ± 10.3	212.6 ± 17.8
***Bsn* cKO**	44	256.8 ± 17.3	300.0 ± 12.2

Confocal stack images (ROIs - region of interest) with 40X objective were taken within the DG supragranular layer. Maximal projection images generated using Image-J software were considered for analysis. DAPI staining was used to mark the area of granule cell layer and cells positive for Calretinin and DCX were counted within this area using cell counter plugin from Image-J. All the values are mean ± SEM.

Table 2: Absolute values for Ki67 staining

Genotype	Total number of sections considered per genotype	Total number of Ki67 positive cells counted per animal
**WT**	25	116.0 ± 7.4
***Bsn* cKO**	25	163.4 ± 11.8

The supragranular layer of the DG was traced based on DAPI staining using a Leica microscope with motorized stage and Neurolucida software. Ki67 positive cells were then marked in this layer throughout the sections. Neuroexplorer software was used to count the Ki67 positive cells. All the values are mean ± SEM.

Online Resource 9. Overview table of antibodies used in this study with specific references.

Primary antibody	Species	Reference
Bassoon	Mouse	Hubler et al., 2012
Tubulin- β	Mouse	Lazarevic et al., 2011
Bassoon	Rabbit	tom Dieck et al., 1998
Calbindin	Rabbit	Dieni et al., 2015
Calretinin	Rabbit	Kobayashi et al., 2014
Ki-67	Rabbit	Clelland et al., 2009
VGAT	Rabbit	Altmüller et al., 2017
VGLUT1	Rabbit	Altmüller et al., 2017
Doublecortin	Goat	Sahay et al., 2011
**Secondary antibody**		
anti-mouse Alexa 488	Donkey	Minter et al., 2017
anti-mouse Cy3	Donkey	Koike et al., 2013
anti-rabbit Alexa 488	Donkey	Tatti et al., 2014
anti-rabbit Cy3	Donkey	Del Cid-Pellitero et al., 2017
anti-goat Cy3	Donkey	Peter et al., 2016
anti-rabbit Alexa 680	Goat	Wang et al., 2016
anti-mouse CF770	Goat	Lucioli et al., 2013
